# What Is Needed to Create a Prosocial Classroom? The Effectiveness of the Dutch Meaningful Roles Program in Children: A Cluster Randomized Controlled Study

**DOI:** 10.1002/jad.70188

**Published:** 2026-06-05

**Authors:** Amanda W. G. van Loon, Tessa M. L. Kaufman

**Affiliations:** ^1^ Child and Adolescent Studies Utrecht University CS Utrecht the Netherlands

**Keywords:** children, classroom‐based intervention, prosocial classroom climate, self‐determination‐theory, well‐being

## Abstract

**Introduction:**

Having a prosocial classroom climate is crucial for promoting children's school and psychological well‐being, as well as in optimizing an effective learning environment. School‐based programs can foster more prosocial classroom climates, but few evidence‐based options are available. This study examined the effectiveness of a primary school program aimed at promoting prosociality by enhancing students' intrinsic motivation through the support of basic psychological needs (autonomy, competence, and relatedness).

**Methods:**

Data stem from 2337 students (46% boys, *M*_age_ = 9.55 years, SD = 1.21) and 225 teachers (76% female, *M*_age_ = 38.69 years, SD = 11.09) across 42 schools in the Netherlands. Schools were randomized to an experimental condition (*N* = 20 schools, *N* = 1199 students, *N* = 115 teachers) or control condition (*N* = 22 schools, *N* = 1138 students, *N* = 110 teachers). Regression analyses were conducted at mid‐ and end‐of‐year, adjusted for baseline levels, comparing conditions in the full sample and a subsample with ideal program fidelity.

**Results:**

Results across the entire sample showed no significant effects. Teachers reported increased attitudes toward stimulating prosocial behavior and autonomy. When the program was implemented as intended (with a subsample of *N* = 202–253 students), small effects were found mainly mid‐school year. Students who received the program fared better than those in the control condition in terms of fulfilling basic psychological needs, prosocial classroom climate, school well‐being and safety, and antisocial behavior.

**Conclusion:**

In conclusion, no effects were found in the full sample, only in a small subgroup that reported ideal program fidelity. Understanding optimal program implementation is essential, as effectiveness depends on the degree of program fidelity.

## 1 Background

To promote children's school and psychological well‐being, and optimize an effective learning environment, a positive prosocial classroom climate is of crucial importance (Evans et al. 2009; Wang et al. 2020). Positive, caring, and supportive classroom climates are associated with less bullying perpetration and victimization (Saarento and Salmivalli 2015; Thornberg et al. 2022). Specifically, prosocial classrooms are characterized by prosocial behavior, collaboration, few conflicts, and high‐quality teacher and peer interactions (Khalfaoui et al. 2021; Villardón‐Gallego et al. 2018; Wang et al. 2020).

Guided by basic psychological needs theory (Ryan and Deci 2017), it is posited that creating a prosocial classroom climate requires strategies that stimulate students' *intrinsic* motivation for positive behaviors, through fulfilling their basic psychological needs (Cheon et al. 2019; Ryan and Deci 2023, 2019). These needs concern *autonomy*, that is, feeling control over one's decisions, *competence*, that is, trusting one's own ability, and *relatedness*, that is, feeling connected and safe. Basic psychological needs fulfillment is associated with greater empathy towards others, better coping with conflicts, internalization of a prosocial classroom norm, a caring classroom environment, and motivation to behave prosocial (Cheon et al. 2019). Moreover, goal‐framing theory subsequently explains *how* such behaviors can persist within *classrooms*: it posits that group norms determine which behaviors are supported and reinforced, versus which behaviors are rejected (Lindenberg and Steg 2007). Therefore, prosocial behavior must become a social *norm* in the classroom to ensure that it persists. Maintaining such a norm occurs through perceiving prosocial attitudes and behavior, as well as support by others (Lindenberg and Steg 2007).

To this end, the current study investigates the effectiveness of a school‐based program promoting a prosocial classroom climate in primary schools, stimulated by increasing prosocial intrinsic motivation through enhancing basic psychological needs (Dutch adaptation of Meaningful Roles; van Loon and Kaufman 2023). The Dutch adaptation of Meaningful Roles was informed by the American pilot version of Meaningful Roles (Ellis et al. 2016): A school‐wide role‐based program, reinforced through the use of compliments, that offers bullies prosocial alternatives to their behavior by increasing autonomy and relatedness. Students in secondary school were assigned roles such as student clerk, announcer, hall monitor, or safety engineer. In addition, students wrote compliments for prosocial behavior among classmates. These compliments were displayed in public. Implementation of the program was associated with significant reductions in all four school‐level factors: fewer fights per month, fewer days absent per month, a lower frequency of illness/injury per month, and less detention per month (Ellis et al. 2016). The current program was adapted for elementary school students (since the intervention developers are experts in implementing social safety programs in primary education) and focuses not specifically on bullies, but on the entire group. Although our framework is broader than the original Meaningful Roles motivational framework, it is still grounded in the same underlying principles. Similar to the original model, our approach builds on the idea that desirable and undesirable behaviors stem from the same fundamental motivations. In our case, these motivations are conceptualized within the framework of the basic psychological needs theory of the self‐determination theory (SDT), emphasizing the need for autonomy, competence, and relatedness, rather than using the original terms referring to dominance or status goals. While we do not explicitly adopt the evolutionary perspective as the underlying framework—since our focus extends beyond bullying—the model remains motivational and functional in nature.

**Figure 1:**
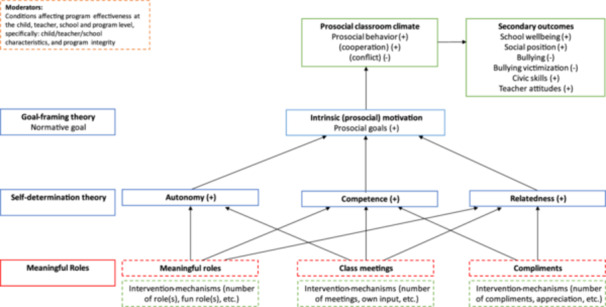
Conceptual model of the research design.

The Dutch Meaningful Roles program is a school‐year approach for grades 5–8, delivered by teachers. The goal of the program is to improve classroom climate and peer relationships in a positive, prosocial way. The program consists of three components: (1) meaningful roles: providing students with meaningful roles to encourage responsibility and cooperation, (2) class meetings: facilitating democratic class meetings to promote mutual understanding and engagement, and (3) compliments: encouraging students to give praise to build connections and self‐confidence. Previous research shows that these (separate) components have positive effects on responsibility, competence, relatedness, interpersonal skills, recognition, prosocial behavior, and problem behavior (Battistich 2005; Edwards and Mullis 2003; Ellis et al. 2016; Embry 2004; Embry and Biglan 2008; Gfroerer et al. 2024). Based on basic psychological needs theory and goal‐framing theory, the expectation is that these three components fulfill students' basic psychological needs, which leads to intrinsic motivation for prosocial behavior, and higher prosocial behavior and a positive classroom climate (e.g., higher school well‐being, less antisocial behavior; see Figure 1 for the conceptual model).

Meta‐analyses of international programs show that school‐wide positive behavior and social and emotional learning (SEL) programs had small to large effects on improving school climate, prosocial behavior, social and emotional skills, positive attitudes, positive self‐esteem, psychological well‐being, citizenship skills, and reduced antisocial behavior in children and adolescents (Charlton et al. 2021; Cipriano et al. 2023; Durlak et al. 2011; Gaffney et al. 2021; Sklad et al. 2012; Tanner‐Smith et al. 2018; Taylor et al. 2017). Research has also demonstrated the effectiveness of intervention programs promoting prosocial behavior in children and adolescents (Caprara et al. 2015; Mesurado et al. 2018; Shin and Lee 2017), specifically for programs that were designed to increase social competence instead of preventing problem behavior (Shin and Lee 2017). Additionally, a meta‐analysis in youth and adults showed that programs aimed to enhance basic psychological needs (i.e., autonomy, competence, and relatedness) were related to increases in basic psychological needs and physical and psychological well‐being (Ntoumanis et al. 2021). Furthermore, previous research in primary and secondary schools that implemented programs related to motivation and satisfaction of basic psychological needs yielded positive effects on basic psychological needs, prosocial behavior, and a positive classroom climate, as well as a reduction in antisocial behavior (Cheon et al. 2019; Manzano‐Sánchez and Valero‐Valenzuela 2019). However, there is a need for (cluster) randomized controlled designs to comprehensively evaluate program effects in diverse, large samples of primary school students.

Addressing these gaps, this study aimed to expand knowledge on school‐based well‐being research in two central ways: (1) by offering empirical support that enhancing basic psychological needs may foster a prosocial classroom climate, and (2) by using a cluster randomized controlled trial (RCT)—a gold standard in effectiveness research (Hariton and Locascio 2018). Specifically, the current study investigated the effectiveness of a school‐based program promoting a prosocial classroom climate (i.e., Dutch adaptation of Meaningful Roles, see Figure 1 for the conceptual model) at two distinct timepoints: mid‐year (T2) and end‐of‐year (T3). We expected (1) fulfillment of basic psychological needs (i.e., autonomy, competence, relatedness), (2) increased intrinsic (prosocial) motivation, (3) an increased prosocial classroom climate (i.e., prosocial behavior, prosocial injunctive norm, more cooperation, less conflict), (4) increased school well‐being and safety, (5) decreased bullying and bullying victimization, (6) increased teacher attitudes about bullying (perception of students), (7) increased teacher attitudes toward stimulating basic psychological need fulfillment, and (8) increased civic skills of students for schools who implemented meaningful roles compared to control schools. As previous research demonstrated that program effects are greater the longer a program is implemented (Huitsing et al. 2020; Limber et al. 2018), we expected more or larger effects at the end of the year (T3) than at mid‐year (T2).

Moreover, we additionally determined the effects of program fidelity—when a program is implemented as *intended*—enabling researchers to draw accurate conclusions about the effectiveness of the program content (Ma et al. 2023; Sanetti et al. 2021). Lack of effectiveness may stem from poor implementation rather than from the program being inherently ineffective (Lane et al. 2004; Sanetti et al. 2021). Indeed, programs that are implemented with higher fidelity tend to yield more positive outcomes, highlighting the role of implementation quality in shaping program success (Durlak et al. 2011; Haataja et al. 2014).

## 2 Methods

### 2.1 Procedure and Design

From January 2023, over 400 schools that implement the KiVa program (KiVa‐schools) were approached to participate in the current study, both in person, by email, and by telephone. We chose to recruit only KiVa schools because Meaningful Roles was developed by the same company that developed the KiVa program. Schools were randomized in April 2023 based on school size (number of participating classes) with an online randomization tool (Research Randomizer 2023) in a ratio of 1:1. A cluster RCT was conducted. After randomization, one control school dropped out due to external circumstances (school reorganization).

From May 2023, schools collected consent for participation in the research study. Schools informed parents and students about the project via a brochure and information letter, containing paper and digital consent forms for parents, students (> 11 years), and teachers. On average, parental consent was 65%, exceeding expectations based on previous studies reporting average parental consent rates between 30% and 60% (Tigges 2003; Wolfenden et al. 2009).

The design consisted of three assessment points. The first assessment was in September‐October 2023 (T1), the second assessment in February–March 2024 (T2), and the final assessment in May–June 2024 (T3). After the first assessment, one intervention school dropped out (both with the implementation of Meaningful Roles and the participation in the study), because they had underestimated the time investment in combination with other developments within the school. The current study is registered in ClinicalTrials (NCT05891067) and the ethical committee of the Faculty of Social Sciences at Utrecht University approved the design of the study (23‐0082) (for the study protocol, see van Loon and Kaufman 2023).

### 2.2 Participants

In total, 46 schools signed up for the study, with three schools dropping out before the randomization. Schools were randomized in an experimental condition (*N* = 20 schools, *N* = 76 classes) or a control condition (*N* = 22 schools, *N* = 78 classes). From the total sample, one control school dropped out after randomization and one intervention school dropped out after the first assessment (*n* = 4 classrooms, T1). There were no school dropouts at T2 or T3.

Supporting Information: Table S1 presents student demographics (*N* = 2,337 students aged 6–13, *M*_age_ = 9.55, *SD* = 1.21; *boy* sex: 48%, gender 46%; *girl* sex 48%, gender 43%; *other* sex 4%, gender 12%). Most students felt connected to Western countries (*N* = 1932, 88%; e.g., the Netherlands, Indonesia, Italy, or France, based on the categorization of Statistics Netherlands; CBS 2025) and others to non‐Western countries (*N* = 264, 12%; e.g., Turkey, Morocco, or Suriname; CBS 2025). Participation across grades was approximately equal (with a slightly larger share in the higher grades; see Supporting Information).

**Figure 2:**
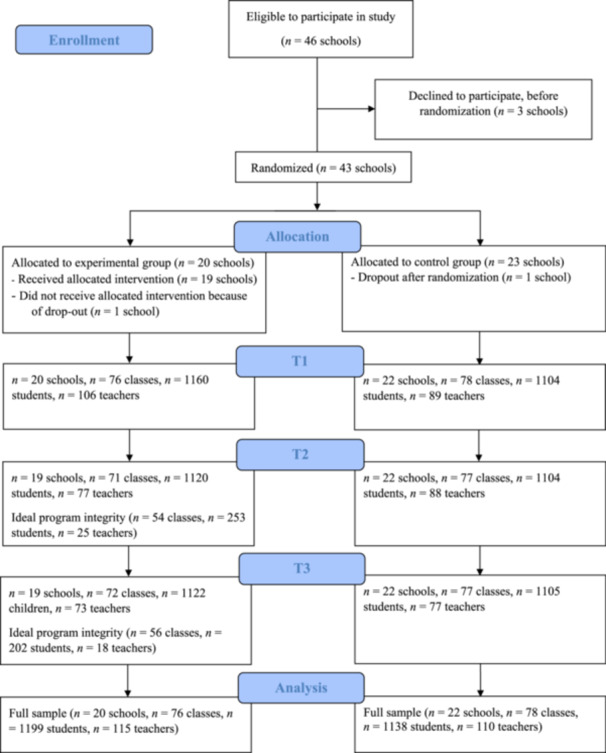
Flow chart of study.

Supporting Information: Table S2 shows teacher demographics (*N* = 225; aged 21–65, *M*_age_ = 38.7, *SD* = 11.1; female sex 77%, gender 76%, 1% preferred not to say). Most teachers felt connected to a Western country (98%). Participation across school years was approximately equal, similar to student reports (see Supporting Information). This study followed the Consolidated Standards of Reporting Trials Guidelines (CONSORT; Begg 1996), as illustrated in Figure 2.

### 2.3 Dutch Meaningful Roles Program

The Dutch adaptation of Meaningful Roles (SterkWerk) is a classroom‐based program implemented by teachers in primary school groups 5 to 8 (students aged 8–12 years). Teachers from intervention schools received two training sessions between August and October 2023 (i.e., two 3‐h sessions), delivered by trained and experienced educational professionals (*N* = 4, each training 3–6 schools). During the training, teachers received information about the program (including the theoretical framework) and how to implement its components in the classroom. For example, teachers received advice on assigning responsibilities (roles) to students instead of assignments (tasks). In addition, the structure of class meetings and how to best facilitate them were discussed, as well as how to encourage and make (giving) compliments visible. The first assessment was conducted as early as possible in the school year, often after the first teacher training (this way the trainer could still remind the school of the consent forms or other steps that had to be taken) and before the second training day. Teachers were only allowed to begin implementing Meaningful Roles after the questionnaires had been administered. Even though there was a training and manual for the intervention program, there was no fully standardized implementation across schools, as teachers could decide for themselves what worked best in their classroom (e.g., letting students write a motivation letter or use a raffle to assign the roles).

The program consists of three main components: meaningful roles, class meetings, and compliments (see Figure 1; van Loon and Kaufman 2023). Regarding the roles, students are assigned specific roles to actively develop autonomy and responsibility, competence, and prosocial behavior. These roles, such as newsreader or plant caretaker, are prosocial in nature because students help one or more peers, contribute to the whole class, or are considerate of other students in the class. It is expected that these activities encourage interaction and, therefore, connectedness. Furthermore, we expect that the roles give students autonomy and responsibility, as students are free to fulfill the role as they see fit. The roles are aimed at maximizing each child's strengths and talents by giving students the experience of contributing something that increases their confidence in their own abilities. During weekly class meetings, students take the lead in discussions about classroom functioning and agreements, discussing their feelings and the classroom atmosphere. It is expected that this promotes interaction and connection with each other and that it fosters students' sense of competence, autonomy, and responsibility in social classroom processes. Finally, students learn how to give and receive effective compliments and practice this on a structured basis, which is expected to strengthen trust in others and self‐confidence. By giving frequent and explicit compliments, emphasizing roles and each other's strengths, students gain appreciation for themselves and others and create a group norm that structurally reinforces prosocial behavior. It is recommended to display the compliments in a visible place in the classroom and to highlight them during class meetings.

### 2.4 Control Condition

The control condition did not receive any materials or training related to Meaningful Roles during the program's implementation in the experimental condition (treatment‐as‐usual). Schools in the control condition were offered the opportunity to receive materials and training after data collection had been completed.

### 2.5 Instruments

Table 1 presents an overview of the instruments at each assessment point. Information on prosocial classroom climate (i.e., prosocial behavior, injunctive norm to behave prosocially, classroom cooperation and conflict), basic psychological needs (i.e., autonomy, competence, relatedness), school well‐being and safety, and antisocial behavior (bullying, bullying perpetration, teacher attitudes about bullying) were reported by students, and teachers additionally provided information on attitudes towards stimulating basic psychological need fulfillment and civic skills of students (see Supporting Material).

**Table 1:** Overview of the outcomes: information on variable names/definitions, instruments, timepoints, answer scales, and source.

Outcome	Variable name (Definition/Example item)	Instrument (#items, reference, reliability)	Timepoint	Answer scale	Source
Primary outcomes					
Prosocial behavior: Individual	Individual prosocial behavior (“I try to be kind to classmates”)	Based on the strengths and difficulties questionnaire (SDQ, Van Widenfelt et al. 2003), subscale prosocial behavior (4 items, *α* = 0.63–0.69 for T1–T3)	T1, T2, T3	1 (never)—4 (always)	Child
Prosocial behavior: Injunctive norm	Group norm (children's attitudes towards prosocial behavior that are generally observed in the classroom; Sentse et al. 2015)	Developed for this study (3 items, *α* = 0.71–0.83 for T1–T3)	T1, T2, T3	1 (not true)—3 (definitely true)	Child
Prosocial behavior: Cooperation	Cooperation in the classroom (“In this classroom, children help each other”)	Classroom Peer Context Questionnaire (CPCQ, Boor‐Klip et al. 2014, 2016, 2017), subscale cooperation (4 items, *α* = 0.74–0.81 for T1–T3)	T1, T2, T3	1 (not true at all)—5 (totally true)	Child
Prosocial behavior: conflict	Conflict in the classroom (“In this classroom, children are mean to each other”)	Classroom Peer Context Questionnaire (CPCQ, Boor‐Klip et al. 2016, 2017), subscale conflict (2 items, *α* = 0.71–0.76 for T1–T3)	T1, T2, T3	1 (not true at all)—5 (totally true)	Child
Intrinsic (prosocial) motivation	Social goals (“I think it is important to help someone”)	Developed for this study, based on a measure of social achievement goals (Rudolph et al. 2011; 4 items, *α* = 0.65–0.71 for T1–T3)	T1, T2, T3	1 (not true)—3 (definitely true)	Child
Autonomy	Social autonomy and responsibility (“In this classroom, you can choose how you want to do something”)	Developed for this study, based on research on *de Vreedzame School* (Battistich et al. 1997; Lickona and Davidson 2003; Pauw 2013) and the Perceived Choice Awareness Scale (Sheldon et al. 2000; 5 items, *α* = 0.67–0.74 for T1–T3)	T1, T2, T3	1 (never)—4 (always)	Child
Competence	Self‐esteem (“I am confident with myself”)	Developed for this study, based on de Rosenberg Self‐Esteem Scale (RSES, Franck et al. 2008; Rosenberg 1965; 5 items, *α* = 0.77–0.81 for T1–T3)	T1, T2, T3	1 (not true)—3 (definitely true)	Child
Relatedness	Comfort and cohesion (“In this classroom, I can be myself”)	Classroom Peer Context Questionnaire (CPCQ, Boor‐Klip 2014, 2016, 2017), subscale comfort and cohesion (6 items, *α* = 0.87–0.89 for T1–T3)	T1, T2, T3	1 (not true at all)—5 (totally true)	Child
Secondary outcomes					
School well‐being	Enjoyment and connectedness with class and school (“I like going to school”)	Based on previous research on KiVa (Kärnä et al. 2011; Kaufman et al. 2021; 7 items, *α* = 0.90–0.91 for T1–T3)	T1, T2, T3	1 (never)—4 (always)	Child
School safety	Safety at school (“Safety at and around school”)	Based on previous research on KiVa (1 item)	T1, T2, T3	1 (very unsafe)—5 (very safe)	Child
Antisocial behavior: Bullying	Bullying (“How often have you bullied other children the past few months?”)	Olweus Bully‐Victim questionnaire (OBVQ, Olweus 1996; 1 item)	T1, T2, T3	1 (never)—5 (multiple times a week)	Child
Antisocial behavior: Bullying victimization	Bullying victimization (“How often have you been bullied the past few months?”; “Others threatened me or forced me to do things I did not want to do”)	Olweus Bully‐Victim questionnaire (OBVQ, Olweus 1996; 1 general item; 7 items, *α* = 0.89–0.88 for T1–T3)	T1, T2, T3	1 (never)—5 (multiple times a week)	Child
Antisocial behavior: Teacher attitudes	Teacher attitudes about bullying (discussing bullying, attitudes towards bullying, and effectiveness in reducing bullying)	Based on previous research on KiVa (Veenstra et al. 2014; 3 separate items)	T1, T2, T3	1 (no/it is something good/nothing) —5 (more than eight times/it is something very bad/very much)	Child
Teacher attitudes	Teacher attitudes towards prosocial behavior, autonomy, competence, and relatedness	Developed for this study (prosocial behavior, 7 items, *α* = 0.80–0.85 for T1–T3; autonomy, five items, α = 0.77–0.78 for T1–T3; competence, 4 items, *α* = 0.82–0.83 for T1–T3; relatedness, 7 items, *α* = 0.84–0.86 for T1–T3)	T1, T2, T3	1–10	Teacher
Civic skills of students	Civic skills (democratic action, socially responsible action, dealing with conflicts, dealing with differences)	Based on the *Vragenlijst Burgerschap Meten* (Daas et al. 2019; 10 items, *α* = 0.81–0.85 for T1–T3)	T1, T2, T3	1 ((almost) never)—4 ((almost) always)	Teacher
Other characteristics					
Child characteristics (demographics)	Demographics (age, sex, gender, ethnic identity, grade, condition)	Developed for this study (9 items)	T1	1 (boy), 2 (girl), 3 (different); 1 (Western), 2 (mix/non‐Western); grades 5–8; 1 (exp), 0 (contr)	Child
Teacher characteristics (demographics)	Demographics (age, sex, gender, ethnic identity, grade, condition)	Developed for this study (7 items)	T1	1 (male), 2 (female), 3 (different); 1 (Western), 2 (mix/non‐Western); 1 (grades 5–6), 2 (grades 6–7), 3 (different); 1 (exp), 0 (contr)	Teacher

### 2.6 Statistical Analyses

Statistical analyses were performed using SPSS and Mplus. We followed an intention‐to‐treat approach, and included all participants in the analyses, including participants of classrooms who did not start the program or did not implement the program (well). We followed this approach to reduce motivation as potential confounding effect and to investigate the effectiveness of the assigned condition (Montori and Guyatt 2001). Before the analyses, to check whether the randomization was successful, possible baseline (T1) differences in demographics and outcome variables (baseline assessment) between students in the intervention program and the control condition were checked with independent *t*‐tests (for continuous variables) and chi‐square analyses (for categorical variables). Moreover, we checked whether there were differences at T1 between participants and dropouts at T2 and T3 by conducting independent *t*‐tests (for continuous variables) and chi‐square analyses (for categorical variables). Furthermore, we performed correlation analyses for all analyzed variables. Students who did not give consent or did not receive parental consent to participate in the study did not complete any research questionnaires (but they did receive the intervention program). Therefore, we have no data on these students, and we could not conduct analyses to investigate whether they differed from participating students.

To investigate the effectiveness of the program, regression analyses were performed in Mplus. Although the program was implemented at the classroom level, individual experiences and engagement varied substantially (e.g., whether students received a role); hence, analyses were conducted at the individual level. The dependent variables were the outcome measures at intermediate (T2) or posttest (T3), condition was the predictor variable (1 = intervention, 0 = control), and baseline (T1), potential demographic differences between the experimental and control condition at T1, and intermediate measures (T2; when dependent variables were the outcome measures at T3) were the covariates. Separate models were run for primary and secondary outcomes at T2 and T3 to account for the interrelationships between effects. The covariance of condition and the specific variable(s) were also included in the model. Including the covariance allows for a more realistic modeling of the data and a better estimate of the regression coefficients (due to the interdependence of the predictors). The regression analyses were clustered at school level and Robust Maximum Likelihood (MLR) was used as an estimator. The MLR is robust against (potential) deviations from normality and nonindependence. In addition, the Full Information Maximum Likelihood (FIML) method was used, a method that accounts for missing values while utilizing all available data. Effect sizes (Cohen's *d*) were calculated using an online program (Lenhard and Lenhard 2022), based on the standardized betas. A positive Cohen's *d* indicates improvements in favor of the intervention condition compared to the control condition. Small effect sizes were considered *d* = 0.20, medium effect sizes *d* = 0.50, and large effect sizes *d* = 0.80 (Cohen 1988).

We conducted sensitivity analyses to determine the impact of program fidelity on program effectiveness. We repeated the regression analyses in a subgroup of students with ideal program fidelity. Furthermore, we investigated possible baseline (T1) differences between the high fidelity, low fidelity, and control condition by conducting ANOVAs. To assess program fidelity, we used student self‐report data on the three main program components, namely meaningful roles, class meetings, and compliments. These data were combined to create one variable for each measurement moment (T2 and T3). Conditions were that students had at least one role in the past months and worked with the role(s) at least once a month (T2 *N* = 751, 68%; T3 *N* = 679, 61%), had at least one class meeting per week (T2 *N* = 560, 51%; T3 *N* = 473, 43%), and received at least one compliment per week (T2 *N* = 657, 59%; T3 *N* = 655, 59%), see Supporting Information: Table S3 for the descriptive statistics (Supporting Material). The subgroup only included students who met all three conditions (T2 *N* = 253, 23%; T3 *N* = 202, 18%). Analyses were also repeated for the low program fidelity subgroup. The confidence intervals of both groups (ideal/high and low program fidelity) were compared to determine whether the subgroups differed from each other.

Regression analyses were repeated in a subgroup of teachers with ideal program fidelity: where teachers indicated that students performed the role(s) (very) independently (T2 *N* = 56, 74%; T3 *N* = 53, 77%), met at least once per week (T2 *N* = 33, 44%; T3 *N* = 25, 36%), and that teachers paid attention to compliments and ensured that compliments were given at least once a week (T2 *N* = 67, 92%; T3 *N* = 63, 93%), see Supporting Information: Table S4 for the descriptive statistics (Supporting Material). The subgroup only included teachers who met all three conditions (T2 *N* = 25, 34%; T3 *N* = 18, 26%). Analyses were also repeated for the low program fidelity subgroup, and the two groups were compared by looking at the confidence intervals. We also investigated possible baseline (T1) differences between the high fidelity, low fidelity and control condition by conducting ANOVAs.

## 3 Results

### 3.1 Randomization, Dropout, and Correlations

Regarding student self‐reports, there were no differences at T1 between the experimental and control condition for demographics (see Supporting Information: Table S1 in the Supporting material). For the study variables (see Table 2), there were differences at T1 between the experimental and control conditions for prosocial behavior (individual and classroom cooperation), safety at school, bullying victimization, and teacher attitudes about discussing bullying. The control condition scored higher on prosocial behavior and bullying victimization, while the experimental condition scored higher on safety at school and teacher attitudes about discussing bullying (Table 2). There were some differences at T1 between participants and dropouts at T2 and T3 (see Supporting Information: Tables S5 and S6 in the Supporting Material). Dropouts more often had a non‐Western ethnic identity, came from lower grades, and reported lower scores on classroom cooperation and higher levels of teacher attitudes toward the effectiveness of reducing bullying (Supporting Information: Tables S5 and S6). There were no other differences at T1 between the experimental and control condition and between participants and dropouts with respect to demographics and study variables (Tables 2, Supporting Information: Tables S5 and S6).

**Table 2:** Descriptives per outcome, differences at T1 between the experimental (*N* = 1160) and control (*N* = 1105) condition, and regression analyses at T2 and T3 for students.l

	Experimental condition^a^			Control condition^a^				Differences at T1			Regression^b^		
	T1	T2	T3	T1	T2	T3		Outcome at T2			Outcome at T3		
	*M* (SD)	*M* (SD)	*M* (SD)	*M* (SD)	*M* (SD)	*M* (SD)	*t* (df)	*β*	*B* (SE)	*d*	*β* (*p*)	*B* (SE)	*d*
Autonomy	2.96 (0.52)	2.94 (0.53)	2.93 (0.55)	2.94 (0.54)	2.92 (0.54)	2.94 (0.55)	−1.00 (2252)	0.01	0.01 (0.04)	0.12	0.02	−0.02 (0.03)	0.14
Competence	2.59 (0.42)	2.59 (0.43)	2.59 (0.43)	2.57 (0.42)	2.58 (0.43)	2.57 (0.45)	−1.11 (2254)	−0.01	−0.01 (0.02)	−0.01	−0.01	0.01 (0.02)	−0.02
Relatedness	4.14 (0.72)	4.05 (0.75)	4.04 (0.76)	4.13 (0.75)	4.06 (0.79)	4.08 (0.78)	−0.32 (2252)	−0.01	−0.02 (0.05)	−0.03	0.02	−0.03 (0.03)	0.13
Intrinsic (prosocial) motivation	2.65 (0.38)	2.65 (0.38)	2.64 (0.38)	2.66 (0.38)	2.64 (0.39)	2.63 (0.42)	0.80 (2256)	0.03	0.02 (0.02)	0.15	−0.01	0.01 (0.02)	−0.02
Prosocial behavior: individual	2.80 (0.51)	2.85 (0.50)	2.84 (0.52)	2.88 (0.51)	2.86 (0.52)	2.88 (0.53)	3.45 (2258)***	0.02	0.02 (0.03)	0.14	0.02	−0.02 (0.03)	0.13
Prosocial behavior: injunctive norm	2.48 (0.49)	2.41 (0.53)	2.36 (0.57)	2.50 (0.51)	2.40 (0.53)	2.37 (0.58)	0.88 (2262)	0.02	0.02 (0.04)	0.14	0.01	−0.01 (0.03)	0.12
Prosocial behavior: cooperation	3.92 (0.59)	3.88 (0.59)	3.87 (0.65)	3.98 (0.62)	3.92 (0.64)	3.95 (0.63)	2.42 (2257)*	−0.02	−0.02 (0.05)	−0.03	0.03	−0.04 (0.03)	0.15
Prosocial behavior: conflict	2.11 (0.85)	2.17 (0.86)	2.18 (0.84)	2.17 (0.87)	2.16 (0.86)	2.13 (0.86)	1.79 (2258)	0.02	0.03 (0.07)	0.14	−0.03	0.06 (0.05)	−0.06
School well‐being	3.18 (0.60)	3.12 (0.62)	3.11 (0.63)	3.15 (0.65)	3.10 (0.65)	3.11 (0.64)	−1.24 (2247)	0.00	0.00 (0.03)	0.00	−0.01	−0.01 (0.03)	−0.02
Safety at school	4.06 (0.89)	4.08 (0.84)	4.08 (0.84)	3.98 (0.97)	4.01 (0.92)	4.05 (0.88)	−2.02 (2248)*	0.02	0.04 (0.05)	0.15	−0.00	−0.01 (0.06)	−0.01
Bullying	1.19 (0.61)	1.18 (0.58)	1.18 (0.59)	1.24 (0.68)	1.20 (0.65)	1.18 (0.59)	1.78 (2172)	−0.01	−0.02 (0.02)	−0.03	0.02	0.02 (0.03)	0.13
Bullying victimization	1.46 (0.99)	1.47 (0.95)	1.39 (0.84)	1.62 (1.10)	1.56 (1.06)	1.47 (0.95)	3.62 (2195)***	−0.02	−0.04 (0.05)	−0.04	−0.02	−0.03 (0.04)	−0.03
Bullying victimization (max)	1.78 (1.19)	1.77 (1.17)	1.62 (1.06)	1.96 (1.28)	1.87 (1.26)	1.78 (1.19)	3.38 (2244)***	−0.01	−0.03 (0.08)	−0.03	−0.05*	−0.10 (0.05)	−0.09
Teacher attitudes: discussing bullying	2.61 (1.19)	2.73 (1.15)	2.60 (1.20)	2.44 (1.21)	2.66 (1.17)	2.45 (1.26)	−3.33 (2234)***	0.01	0.02 (0.11)	0.12	0.04	0.09 (0.09)	0.17
Teacher attitudes: attitudes toward bullying	4.48 (0.95)	4.61 (0.76)	4.63 (0.76)	4.46 (0.95)	4.56 (0.84)	4.56 (0.84)	−0.54 (2235)	0.03	0.04 (0.04)	0.14	0.04	0.06 (0.04)	0.18
Teacher attitudes: effectiveness reducing bullying	3.53 (1.04)	3.34 (1.01)	3.20 (1.09)	3.45 (1.09)	3.41 (1.07)	3.24 (1.14)	1.91 (2209)	−0.05	−0.10 (0.06)	−0.10	−0.01	−0.03 (0.06)	−0.03

*Note:* Number of participants varies per variable and assessment (experimental condition *N* = 1113–1160; control condition *N* = 1086–1105).

^a^ Condition is coded as control condition = 0 and experimental condition = 1.

^b^ Corrected for clustering at school level and for interdependence between effects.

*p < 0.05; ***p < 0.001.

Regarding teachers' self‐reports, there were differences at T1 between the experimental and control condition for sex and gender (see Supporting Information: Table S2 in the Supporting Material). There were fewer women in the control condition (for both sex and gender; Supporting Information: Table S2). Hence, gender was included in the regression analyses for teachers. For the study variables there were no differences at T1 between the experimental and control condition, except for teacher attitudes towards autonomy (see Table 3). Teachers in the control condition had higher levels of attitudes towards autonomy compared to teachers in the intervention condition (Table 3). There were no differences at T1 between participants and dropouts at T2 and T3 study variables, only for sex and condition (see Supporting Information: Table S7 in the Supporting Material). Dropouts were more often male (sex) and came from the experimental condition (Supporting Information: Table S7).

**Table 3:** Descriptives per outcome, differences at T1 between the experimental (*N* = 100) and control (*N* = 90) condition, and regression analyses at T2 and T3 for teachers.

	Experimental condition^a^			Control condition^a^				Differences at T1			Regression^b^		
	T1	T2	T3	T1	T2	T3		Outcome at T2			Outcome at T3		
	*M* (SD)	*M* (SD)	*M* (SD)	*M* (SD)	*M* (SD)	*M* (SD)	*t* (df)	*β*	*B* (SE)	*d*	*β*	*B* (SE)	*d*
Teacher attitudes: prosocial behavior	7.79 (0.68)	7.86 (0.79)	8.15 (0.73)	8.00 (0.86)	8.07 (0.72)	8.07 (0.74)	1.86 (188)	−0.13	−0.181 (0.127)	−0.25	0.14*	0.20 (0.09)	0.39
Teacher attitudes: autonomy	7.61 (0.86)	7.86 (0.85)	8.23 (0.79)	8.05 (0.87)	8.04 (0.73)	8.12 (0.71)	3.51 (188)***	0.03	0.042 (0.114)	0.16	0.14*	0.20 (0.09)	0.38
Teacher attitudes: competence	8.02 (0.82)	8.02 (0.81)	8.29 (0.81)	8.16 (0.91)	8.11 (0.76)	8.30 (0.69)	1.11 (185)	−0.06	−0.092 (0.101)	−0.12	0.06	0.09 (0.07)	0.23
Teacher attitudes: relatedness	8.25 (0.75)	8.25 (0.81)	8.38 (0.74)	8.43 (0.81)	8.33 (0.73)	8.44 (0.71)	1.54 (185)	−0.06	−0.087 (0.110)	−0.12	−0.02	−0.03 (0.10)	−0.04
Civic skills of students	3.03 (0.39)	3.06 (0.41)	3.09 (0.39)	3.02 (0.35)	3.05 (0.33)	3.03 (0.36)	−0.18 (168)	0.00	0.001 (0.050)	0.10	0.09	0.07 (0.05)	0.28

*Note:* Number of participants varies per variable and assessment (experimental condition *N* = 73–100, control condition *N* = 77–90). Gender is included as a covariate in all analyses (only the categories male and female).

^a^ Condition is coded as control condition = 0 and experimental condition = 1.

^b^ Corrected for clustering at school level and interdependence between effects.

*p < 0.05; ***p < 0.001.

Supporting Information: Tables S8–S12 in the Supporting Material present the correlation analyses for primary and secondary outcomes. In general, across all three timepoints (T1–T3), basic psychological needs, prosocial behavior, well‐being, and safety at school are positively correlated, while conflict, bullying, and bullying victimization are negatively associated with these outcomes. Conflict, bullying, and bullying victimization are positively related to each other (Supporting Information: Tables S8–S12). Regarding teacher attitudes, discussing bullying shows inconsistent results across timepoints (i.e., positively linked to positive outcomes such as autonomy and prosocial behavior, but also to bullying and victimization). Attitudes toward bullying are positively related to positive outcomes (e.g., basic psychological needs) and negatively with conflict, bullying, and victimization. Attitudes regarding the effectiveness of reducing bullying correlates positively with positive outcomes but shows no significant relationship with bullying and bullying victimization (Supporting Information: Tables S9–S12).

Stability coefficients (calculated based on the correlations per variable from T1–T2 and T2–T3 in the whole group) for the primary outcomes ranged from 0.46 to 0.71, indicating moderate to high stability (autonomy and prosocial behavior: injunctive norm are least stable, relatedness is most stable). Stability coefficients for the secondary outcomes ranged from 0.36 to 0.67, indicating moderate to high stability (bullying and teacher attitudes towards bullying and discussing bullying are least stable, well‐being is most stable).

### 3.2 Program Effectiveness in Full Sample

First, it was investigated to what extent there were effects when the entire sample, irrespective of the level of program fidelity, was considered. As presented in Table 2, there were no significant effects on any of the outcome measures, except for bullying victimization (maximum score) at T3. Because the effect size was very low (*d* = 0.09), we interpret this as no meaningful effect.

Table 3 presents the results of the regression analyses for teachers. Results showed no significant effect for students' civic skills, at either T2 or T3. For teacher attitudes towards stimulating basic psychological needs at T3, significant program effects were found for stimulating prosocial behavior and autonomy. The effects were small to medium (Cohen 1988).

### 3.3 Program Effectiveness for Ideal Program Fidelity

For analyses at T2, differences at T1 between the high fidelity, low fidelity, and control conditions were found for all variables except for teacher attitudes toward the effectiveness of reducing bullying (see Table 4A). Differences at T1 between high and low fidelity were observed for autonomy, competence, relatedness, intrinsic motivation, prosocial behavior (individual, injunctive norm, cooperation, conflict), school well‐being, bullying, bullying victimization, and teacher attitudes towards bullying. Differences at T1 between high fidelity and the control condition were observed for autonomy, competence, relatedness, prosocial behavior (individual, conflict), school well‐being, safety, bullying, bullying victimization, and teacher attitudes towards bullying. Finally, differences at T1 between low fidelity and the control condition were observed for prosocial behavior (individual, injunctive norm, cooperation), and teacher attitudes about discussing bullying (Table 4A). In general, the most positive scores were observed for the high fidelity condition, followed by the control condition and the low fidelity condition.

**Table 4A:** Sensitivity analyses for program fidelity at T2: Descriptives and differences at T1 between the experimental (high fidelity: *N* = 253, Low fidelity: *N* = 855) and the control (*N* = 1104) condition for students.

	Experimental condition (high fidelity)		Experimental condition (low fidelity)		Control condition		
	T1	T2	T1	T2	T1	T2	Differences at T1
	*M* (SD)	*M* (SD)	*M* (SD)	*M* (SD)	*M* (SD)	*M* (SD)	*F*‐value^a^
Autonomy	3.09 (0.47)	3.14 (0.47)	2.92 (0.53)	2.88 (0.53)	2.94 (0.54)	2.92 (0.54)	10.99*** (a, b)
Competence	2.67 (0.36)	2.68 (0.34)	2.58 (0.42)	2.56 (0.44)	2.57 (0.42)	2.58 (0.43)	6.20** (a, b)
Relatedness	4.32 (0.58)	4.30 (0.59)	4.08 (0.74)	3.97 (0.77)	4.13 (0.75)	4.06 (0.79)	10.95*** (a, b)
Intrinsic (prosocial) motivation	2.71 (0.33)	2.76 (0.31)	2.63 (0.39)	2.63 (0.39)	2.66 (0.38)	2.64 (0.39)	4.65** (a)
Prosocial behavior: individual	2.96 (0.46)	3.02 (0.44)	2.76 (0.50)	2.80 (0.51)	2.88 (0.51)	2.86 (0.52)	21.37*** (a, b, c)
Prosocial behavior: injunctive norm	2.58 (0.44)	2.51 (0.53)	2.45 (0.50)	2.39 (0.53)	2.50 (0.51)	2.40 (0.53)	8.11*** (a, c)
Prosocial behavior: cooperation	4.07 (0.53)	4.08 (0.52)	3.88 (0.61)	3.81 (0.60)	3.98 (0.62)	3.92 (0.64)	11.61*** (a, c)
Prosocial behavior: conflict	1.98 (0.79)	1.94 (0.78)	2.15 (0.87)	2.24 (0.87)	2.17 (0.87)	2.16 (0.86)	4.95* (a, b)
School well‐being	3.40 (0.47)	3.34 (0.52)	3.11 (0.62)	3.05 (0.64)	3.15 (0.65)	3.10 (0.65)	22.11*** (a, b)
Safety at school	4.16 (0.87)	4.26 (0.71)	4.03 (0.88)	4.03 (0.88)	3.98 (0.97)	4.01 (0.92)	3.95* (b)
Bullying	1.05 (0.24)	1.13 (0.51)	1.23 (0.67)	1.19 (0.61)	1.24 (0.68)	1.20 (0.65)	9.51*** (a, b)
Bullying victimization	1.23 (0.79)	1.31 (0.80)	1.52 (1.02)	1.52 (0.99)	1.62 (1.10)	1.56 (1.06)	13.94*** (a, b)
Bullying victimization (max)	1.52 (0.98)	1.55 (0.97)	1.85 (1.22)	1.84 (1.22)	1.96 (1.28)	1.87 (1.26)	12.62*** (a, b)
Teacher attitudes: discussing bullying	2.62 (1.14)	2.83 (1.11)	2.59 (1.19)	2.70 (1.16)	2.44 (1.21)	2.66 (1.17)	4.69** (c)
Teacher attitudes: attitudes toward bullying	4.64 (0.75)	4.73 (0.58)	4.44 (0.98)	4.58 (0.82)	4.46 (0.95)	4.56 (0.84)	4.57* (a, b)
Teacher attitudes: effectiveness in reducing bullying	3.58 (0.97)	3.37 (0.91)	3.49 (1.06)	3.33 (1.04)	3.45 (1.09)	3.41 (1.07)	1.86

*Note:* Number of participants varies per variable and assessment (experimental condition high fidelity *N* = 252–253, low fidelity *N* = 825–855; control condition *N* = 1096–1104).

^a^ Significant difference between high and low fidelity (a), significant difference between high fidelity and control condition (b), significant difference between low fidelity and control condition (c).

*p < 0.05; **p < 0.01; ***p < 0.001.

When the analyses were performed with a subsample of students who experienced the program being implemented as intended (high fidelity; see Table 4B), the program showed significant positive effects at T2 for basic psychological needs (autonomy, competence, and relatedness), intrinsic (prosocial) motivation, prosocial behavior (individual behavior, cooperation and conflict in the classroom), school well‐being, safety at school, bullying victimization (mean and maximum), and teacher attitudes toward bullying. No significant effects were found for the group norm (injunctive norm), bullying behavior, and teacher attitudes regarding discussing bullying and effectiveness in reducing bullying (Table 4B). The effects for all outcomes were small (Cohen 1988). Differences were found between high and low fidelity for autonomy, relatedness, intrinsic (prosocial) motivation, individual prosocial behavior, classroom cooperation, school well‐being, and school safety, but not for competence, conflict in the classroom, bullying victimization, and teacher attitudes toward bullying (Table 4B).

**Table 4B:** Sensitivity analyses for program fidelity at T2: Effects of the intervention dependent of program fidelity low (*N* = 855) versus high (*N* = 253) compared to the control condition (*N* = 1104).

	Low (vs. control)^a^				High (vs. control)^a^				
	*β*	*B* (SE)	CI (95%)	*d*	*β*	*B* (SE)	CI (95%)	*d*	Difference^b^
Autonomy	−0.04	−0.04 (0.04)	−0.12 to 0.03	−0.08	0.14***	0.19 (0.03)	0.13–0.25	0.39	Yes
Competence	−0.03	−0.02 (0.02)	−0.07 to 0.02	−0.05	0.06*	0.06 (0.03)	0.02–0.11	0.22	No
Relatedness	−0.05	−0.07 (0.05)	−0.18 to 0.03	−0.10	0.11**	0.20 (0.06)	0.08–0.31	0.32	Yes
Intrinsic (prosocial) motivation	−0.01	−0.01 (0.03)	−0.05 to 0.04	−0.01	0.12***	0.11 (0.03)	0.06–0.16	0.33	Yes
Prosocial behavior: individual	−0.01	−0.01 (0.03)	−0.07 to 0.05	−0.02	0.11**	0.14 (0.04)	0.06–0.21	0.38	Yes
Prosocial behavior: injunctive norm	−0.00	−0.00 (0.04)	−0.07 to 0.07	−0.01	0.07	0.10 (0.05)	0.01–0.19	0.24	No
Prosocial behavior: cooperation	−0.06	−0.07 (0.05)	−0.17 to 0.04	−0.11	0.11**	0.17 (0.05)	0.07–0.27	0.33	Yes
Prosocial behavior: conflict	0.05	0.09 (0.07)	−0.05 to 0.23	0.21	−0.08*	−0.18 (0.08)	−0.34 to −0.02	−0.17	No
School well‐being	−0.02	−0.03 (0.03)	−0.09 to 0.03	−0.05	0.08**	0.13 (0.03)	0.07–0.19	0.27	Yes
Safety at school	0.00	0.00 (0.05)	−0.09 to 0.09	0.10	0.11**	0.25 (0.06)	0.14–0.36	0.33	Yes
Bullying	−0.01	−0.01 (0.03)	−0.06 to 0.04	−0.02	−0.03	−0.04 (0.04)	−0.11 to 0.03	−0.05	No
Bullying victimization	−0.00	−0.01 (0.05)	−0.11 to 0.09	−0.01	−0.08*	−0.19 (0.07)	−0.33 to −0.06	−0.16	No
Bullying victimization (max)	0.00	0.01 (0.08)	−0.14 to 0.16	0.11	−0.09*	−0.26 (0.10)	−0.46 to −0.06	−0.17	No
Teacher attitudes: discussing bullying	−0.00	−0.01 (0.12)	−0.24 to 0.23	−0.00	0.03	0.09 (0.09)	−0.09 to 0.28	0.29	No
Teacher attitudes: attitudes toward bullying	0.01	0.02 (0.04)	−0.07 to 0.10	0.12	0.08**	0.16 (0.04)	0.09 to 0.24	0.26	No
Teacher attitudes: effectiveness reducing bullying	−0.05	−0.10 (0.06)	−0.22 to 0.02	−0.09	−0.03	−0.08 (0.07)	−0.22 to 0.06	−0.17	No

*Note:* Number of participants varies per variable and assessment (experimental condition high fidelity *N* = 252–253, low fidelity *N* = 825–855; control condition *N* = 1096–1104).

^a^ Condition is coded as control condition = 0 en experimental condition = 1. Corrected for clustering at the school level and interdependence between effects.

^b^ Significant difference between effect high (high vs. control) and low (low vs. control).

*p < 0.05; **p < 0.01; ***p < 0.001.

For analyses at T3, differences at T1 between the high fidelity, low fidelity, and control condition were found for all variables except for competence, intrinsic motivation, bullying, and teacher attitudes towards bullying and the effectiveness of reducing bullying (see Table 5A). Differences at T1 between high and low fidelity were observed for autonomy, relatedness, prosocial behavior (individual, injunctive norm, cooperation), school well‐being, and bullying victimization. Differences at T1 between high fidelity and the control condition were observed for autonomy, relatedness, conflict, school well‐being, safety, bullying victimization, and teacher attitudes about discussing bullying. Finally, differences at T1 between low fidelity and the control condition were observed for prosocial behavior (individual, cooperation), bullying victimization, and teacher attitudes about discussing bullying (Table 5A). In general, the most positive scores were observed for the high fidelity condition, followed by the control condition and the low fidelity condition.

**Table 5A:** Sensitivity analyses program fidelity at T3: Descriptives and differences at T1 between the experimental (high fidelity *N* = 202; low fidelity *N* = 909) and control (*N* = 1105) condition for students.

	Experimental condition (high fidelity)^a^			Experimental condition (low fidelity)^a^			Control condition^a^			
	T1	T2	T3	T1	T2	T3	T1	T2	T3	Differences at T1
	*M* (SD)	*M* (SD)	*M* (SD)	*M* (SD)	*M* (SD)	*M* (SD)	*M* (SD)	*M* (SD)	*M* (SD)	*F*‐valuea
Autonomy	3.05 (0.49)	3.07 (0.51)	3.10 (0.49)	2.94 (0.52)	2.90 (0.53)	2.89 (0.56)	2.94 (0.54)	2.92 (0.54)	2.94 (0.55)	4.28* (a, b)
Competence	2.64 (0.37)	2.65 (0.35)	2.68 (0.36)	2.58 (0.42)	2.57 (0.44)	2.57 (0.45)	2.57 (0.42)	2.58 (0.43)	2.57 (0.45)	2.27
Relatedness	4.31 (0.58)	4.23 (0.66)	4.26 (0.63)	4.09 (0.74)	4.01 (0.77)	3.99 (0.77)	4.13 (0.75)	4.06 (0.79)	4.08 (0.78)	7.07*** (a, b)
Intrinsic (prosocial) motivation	2.70 (0.31)	2.71 (0.34)	2.69 (0.33)	2.63 (0.40)	2.64 (0.39)	2.63 (0.39)	2.66 (0.38)	2.64 (0.39)	2.63 (0.42)	2.74
Prosocial behavior: individual	2.91 (0.49)	2.98 (0.49)	2.98 (0.47)	2.78 (0.50)	2.82 (0.50)	2.81 (0.53)	2.88 (0.51)	2.86 (0.52)	2.88 (0.53)	10.05*** (a, c)
Prosocial behavior: injunctive norm	2.58 (0.43)	2.51 (0.51)	2.49 (0.52)	2.46 (0.50)	2.39 (0.54)	2.34 (0.58)	2.50 (0.51)	2.40 (0.53)	2.37 (0.58)	5.18** (a)
Prosocial behavior: cooperation	4.08 (0.53)	4.06 (0.52)	4.05 (0.56)	3.88 (0.60)	3.83 (0.60)	3.83 (0.66)	3.98 (0.62)	3.92 (0.64)	3.95 (0.63)	11.24*** (a, c)
Prosocial behavior: conflict	2.00 (0.84)	2.03 (0.85)	2.04 (0.86)	2.14 (0.86)	2.21 (0.85)	2.22 (0.83)	2.17 (0.87)	2.16 (0.86)	2.13 (0.86)	3.23* (b)
School well‐being	3.34 (0.51)	3.27 (0.60)	3.27 (0.56)	3.14 (0.62)	3.08 (0.63)	3.07 (0.64)	3.15 (0.65)	3.10 (0.65)	3.11 (0.64)	8.32*** (a, b)
Safety at school	4.18 (0.88)	4.18 (0.72)	4.18 (0.76)	4.03 (0.89)	4.05 (0.88)	4.06 (0.85)	3.98 (0.97)	4.01 (0.92)	4.05 (0.87)	3.82* (b)
Bullying	1.17 (0.58)	1.14 (0.52)	1.14 (0.52)	1.20 (0.62)	1.18 (0.59)	1.19 (0.61)	1.24 (0.68)	1.20 (0.65)	1.18 (0.59)	1.72
Bullying victimization	1.27 (0.71)	1.37 (0.83)	1.31 (0.74)	1.49 (1.02)	1.50 (0.99)	1.41 (0.86)	1.62 (1.10)	1.56 (1.06)	1.47 (0.95)	10.43*** (a, b, c)
Bullying victimization (max)	1.61 (1.02)	1.66 (1.05)	1.48 (0.85)	1.81 (1.21)	1.81 (1.20)	1.65 (1.10)	1.96 (1.28)	1.87 (1.26)	1.78 (1.19)	7.92*** (b, c)
Teacher attitudes: discussing bullying	2.73 (1.26)	2.89 (1.15)	2.90 (1.22)	2.59 (1.17)	2.69 (1.15)	2.53 (1.18)	2.44 (1.21)	2.66 (1.17)	2.45 (1.26)	6.76** (b, c)
Teacher attitudes: attitudes toward bullying	4.59 (0.89)	4.66 (0.69)	4.73 (0.63)	4.45 (0.96)	4.60 (0.80)	4.61 (0.78)	4.46 (0.95)	4.56 (0.84)	4.56 (0.84)	1.82
Teacher attitudes: effectiveness reducing bullying	3.57 (1.05)	3.36 (1.04)	3.30 (1.08)	3.50 (1.04)	3.33 (1.01)	3.17 (1.14)	3.45 (1.09)	3.41 (1.07)	3.24 (1.14)	1.57

*Note:* Number of participants varies per variable and assessment (experimental condition high fidelity *N* = 193–202, low fidelity *N* = 875–909; control condition *N* = 1086–1105).

^a^ Significant difference between high and low fidelity (a), significant difference between high fidelity and control condition (b), significant difference between low fidelity and control condition (c).

*p < 0.05; **p < 0.01; ***p < 0.001.

Significant positive effects were found at T3 for competence, relatedness, bullying victimization, and teacher attitudes toward bullying and discussing bullying (see Table 5B). The effects for all outcomes were small (Cohen 1988). Differences were found between high and low fidelity for relatedness and bullying victimization, not for competence and teacher attitudes (Table 5B).

**Table 5B:** Sensitivity analyses program fidelity at T3: Effects of the intervention dependent of program fidelity low (*N* = 909) versus high (*N* = 202) compared to the control condition (*N* = 1105).

	Low (vs control)^a^				High (vs. control)^a^				
	*β*	*B* (SE)	CI (95%)	*d*	*β*	*B* (SE)	CI (95%)	*d*	Difference^b^
Primary outcomes									
Autonomy	−0.05	−0.05 (0.03)	−0.11 to 0.02	−0.09	0.08	0.12 (0.06)	0.00–0.23	0.26	No
Competence	−0.00	−0.00 (0.02)	−0.03 to 0.03	−0.00	0.05*	0.06 (0.02)	0.02–0.10	0.20	No
Relatedness	−0.04	−0.05 (0.03)	−0.11 to 0.01	−0.07	0.05*	0.11 (0.04)	0.03–0.19	0.21	Yes
Intrinsic (prosocial) motivation	0.01	0.00 (0.02)	−0.03 to 0.04	0.11	0.03	0.03 (0.04)	−0.04 to 0.10	0.16	No
Prosocial behavior: individual	−0.03	−0.03 (0.03)	−0.08 to 0.02	−0.07	0.04	0.05 (0.05)	−0.05–0.15	0.17	No
Prosocial behavior: injunctive norm	−0.02	−0.03 (0.03)	−0.09 to 0.04	−0.05	0.05	0.08 (0.04)	−0.01 to 0.16	0.20	No
Prosocial behavior: cooperation	−0.05	−0.06 (0.03)	−0.12 to 0.00	−0.10	0.04	0.07 (0.06)	−0.05 to 0.18	0.18	No
Prosocial behavior: conflict	0.04	0.07 (0.05)	−0.02 to 0.17	0.19	−0.02	−0.05 (0.08)	−0.21 to 0.11	−0.04	No
*Secondary outcomes*									
School well‐being	−0.02	−0.02 (0.03)	−0.07 to 0.03	−0.04	0.03	0.06 (0.041)	−0.02 to 0.14	0.17	No
Safety at school	−0.01	−0.02 (0.06)	−0.12 to 0.09	−0.02	0.03	0.08 (0.08)	−0.08 to 0.23	0.17	No
Bullying	0.02	0.02 (0.03)	−0.03 to 0.08	0.14	−0.01	−0.02 (0.05)	−0.11 to 0.07	−0.03	No
Bullying victimization	−0.01	−0.02 (0.04)	−0.10 to 0.05	−0.03	−0.03	−0.08 (0.05)	−0.17 to 0.02	−0.06	No
Bullying victimization (max)	−0.04	−0.08 (0.05)	−0.18 to 0.02	−0.07	−0.07**	−0.20 (0.05)	−0.31 to −0.10	−0.14	Yes
Teacher attitudes: discussing bullying	0.02	0.04 (0.10)	−0.15 to 0.22	0.13	0.08*	0.28 (0.11)	0.06–0.50	0.27	No
Teacher attitudes: attitudes toward bullying	0.03	0.05 (0.04)	−0.04 to 0.13	0.16	0.07**	0.14 (0.04)	0.07–0.22	0.23	No
Teacher attitudes: effectiveness reducing bullying	−0.02	−0.05 (0.06)	−0.17 to 0.07	−0.04	0.02	0.05 (0.09)	−0.11 to 0.22	0.13	No

*Note:* Number of participants varies per variable and assessment (experimental condition high fidelity *N* = 193–202, low fidelity *N* = 875–909; control condition *N* = 1086–1105).

^a^ Condition is coded as control condition = 0 and experimental condition = 1. Corrected for clustering at the school level and interdependence between effects.

^b^ Significant difference between effect high (high vs. control) and low (low vs. control).

*p < 0.05; **p < 0.01; ***p < 0.001.

For teachers, there was only a difference at T1 between the high fidelity and control condition for teacher attitudes towards autonomy (for analyses at T2 and T3; see Tables 6 and 7A). Teachers in the high fidelity condition reported lower scores on attitudes towards autonomy than teachers in the control condition (Tables 6 and 7A). Regarding teachers' self‐reports, teachers who reported that the program was implemented as intended showed no positive effects for teacher attitudes towards stimulating basic psychological need fulfillment and students' civic skills, at either T2 or T3. There were also no differences between high and low fidelity (Tables 6 and 7B).

**Table 6:** Sensitivity analyses program fidelity at T2: Differences at T1 between the experimental (low fidelity *N* = 49, high fidelity = 25) and control (*N* = 86) condition, and regression analyses for teachers.

	Experimental condition (high fidelity)^a^		Experimental condition (low fidelity)^a^		Control condition^a^		Differences at T1	Regression (low vs. control)				Regression (high vs. control)				
	T1	T2	T1	T2	T1	T2		Outcome at T2				Outcome at T2				
	*M* (SD)	*M* (SD)	*M* (SD)	*M* (SD)	*M* (SD)	*M* (SD)	*F*‐value^b^	*β*	*B* (SE)	CI (95%)	*d*	*β*	*B* (SE)	CI (95%)	*d*	*Diffs* ^c^
Teacher attitudes: prosocial behavior	7.79 (0.70)	7.87 (0.61)	7.85 (0.76)	7.88 (0.87)	8.00 (0.86)	8.07 (0.72)	0.86	−0.11	−0.17 (0.15)	−0.47 to 0.13	−0.23	−0.08	−0.16 (0.12)	−0.39 to 0.07	−0.17	No
Teacher attitudes: autonomy	7.38 (1.05)	7.95 (0.93)	7.75 (0.79)	7.80 (0.82)	8.05 (0.87)	8.04 (0.73)	5.78** (b)	−0.07	−0.11 (0.13)	−0.35 to 0.14	−0.13	0.14	0.27 (0.17)	−0.07 to 0.61	0.39	No
Teacher attitudes: competence	7.76 (1.01)	7.93 (0.77)	8.10 (0.81)	8.05 (0.85)	8.16 (0.91)	8.11 (0.76)	1.76	−0.06	−0.10 (0.14)	−0.37 to 0.18	−0.12	−0.06	−0.11 (0.16)	−0.43 to 0.21	−0.11	No
Teacher attitudes: relatedness	8.27 (0.89)	8.54 (0.70)	8.31 (0.67)	8.15 (0.83)	8.43 (0.81)	8.33 (0.73)	0.53	−0.16*	−0.25 (0.12)	−0.48 to −0.01	−0.32	0.12	0.22 (0.13)	−0.05 to 0.48	0.34	No
Civic skills of students	3.10 (0.36)	3.18 (0.37)	3.01 (0.45)	3.02 (0.42)	3.02 (0.35)	3.05 (0.33)	0.53	−0.02	−0.02 (0.05)	−0.11 to 0.07	−0.05	0.09	0.08 (0.08)	−0.11 to 0.23	0.28	No

*Note:* Number of participants varies per variable and assessment (experimental condition high fidelity *N* = 22–25, low fidelity *N* = 40–49, and control condition *N* = 87–90). Gender is included as covariate in all analyses (only with categories male and female).

^a^ Condition is coded as control condition = 0 and experimental condition = 1

^b^ Significant difference between high and low fidelity (a), significant difference between high fidelity and control condition (b), significant difference between low fidelity and control condition (c).

^c^ Significant difference between effect high (high vs. control) and low (low vs. control).

*p < 0.05; **p < 0.01.

**Table 7A:** Sensitivity analyses program fidelity at T3: Differences at T1 between the experimental (high fidelity *N* = 18, low fidelity *N* = 51) and control (*N* = 90) condition, and regression analyses for teachers.

	Experimental condition (high fidelity)			Experimental condition (low fidelity)			Control condition			Differences at T1
	T1	T2	T3	T1	T2	T3	T1	T2	T3	
	*M* (SD)	*M* (SD)	*M* (SD)	*M* (SD)	*M* (SD)	*M* (SD)	*M* (SD)	*M* (SD)	*M* (SD)	*F*‐value
Teacher attitudes: prosocial behavior	7.79 (0.47)	7.95 (0.64)	8.24 (0.65)	7.81 (0.78)	7.97 (0.75)	8.12 (0.78)	8.00 (0.86)	8.07 (0.72)	8.07 (0.74)	0.86
Teacher attitudes: autonomy	7.64 (0.65)	8.28 (0.68)	8.46 (0.66)	7.62 (0.97)	7.84 (0.88)	8.18 (0.83)	8.05 (0.87)	8.04 (0.73)	8.12 (0.71)	5.78**
Teacher attitudes: competence	7.83 (0.81)	7.88 (0.81)	8.26 (0.72)	8.05 (0.97)	8.18 (0.87)	8.31 (0.86)	8.16 (0.91)	8.11 (0.76)	8.30 (0.69)	1.76
Teacher attitudes: relatedness	8.08 (0.62)	8.55 (0.69)	8.46 (0.66)	8.39 (0.77)	8.30 (0.84)	8.40 (0.76)	8.43 (0.81)	8.33 (0.73)	8.44 (0.71)	0.53
Civic skills of students	3.02 (0.40)	3.09 (0.47)	3.15 (0.43)	3.02 (0.40)	3.04 (0.39)	3.05 (0.38)	3.02 (0.35)	3.05 (0.33)	3.03 (0.36)	0.53

*Note:* Number of participants varies per variable and assessment (experimental condition high fidelity *N* = 16–18, low fidelity *N* = 43–51, control condition *N* = 77–90).

**p < 0.01.

**Table 7B:** Sensitivity analyses program fidelity at T3: Effects of the intervention dependent of program fidelity low (*N* = 51) versus high (*N* = 18) compared to the control condition (*N* = 90).

	Regression (low vs. control)^a^				Regression (high vs. control)^a^				
	Outcome at T3				Outcome at T3				
	*β*	*B* (SE)	CI (95%)	*d*	*β*	*B* (SE)	CI (95%)	*d*	Difference^b^
Teacher attitudes: prosocial behavior	0.10	0.15 (0.12)	−0.07 to 0.38	0.31	0.08	0.16 (0.12)	−0.08 to 0.41	0.26	No
Teacher attitudes: autonomy	0.11	0.16 (0.10)	−0.03 to 0.36	0.32	0.09	0.18 (0.17)	−0.15 to 0.51	0.27	No
Teacher attitudes: competence	0.04	0.06 (0.08)	−0.10 to 0.22	0.18	0.03	0.05 (0.14)	−0.22 to 0.32	0.15	No
Teacher attitudes: relatedness	0.00	0.00 (0.10)	−0.19 to 0.19	0.00	−0.02	−0.04 (0.18)	−0.39 to 0.31	−0.04	No
Civic skills of students	0.04	0.03 (0.05)	−0.07 to 0.14	0.17	0.08	0.09 (0.06)	−0.03 to 0.21	0.27	No

*Note:* Number of participants varies per variable and assessment (experimental condition high fidelity *N* = 16–18, low fidelity *N* = 43–51, control condition *N* = 77–90). Gender is included as covariate in all analyses (only with categories male and female).

^a^ Condition is coded as control condition = 0 and experimental condition = 1.

^b^ Significant difference between effect high (high vs. control) and low (low vs. control).

## 4 Discussion

A positive, prosocial classroom climate is of crucial importance to optimize an effective learning environment, and to promote children's school and psychological well‐being (Evans et al. 2009; Wang et al. 2020). School‐based interventions may be an ideal approach to achieve this, but knowledge is lacking on the effectiveness of programs based on basic psychological needs theory and (cluster) RCT designs to evaluate such effects. To address this gap, the current study examined the effectiveness of the Dutch Meaningful Roles—a classroom‐based intervention designed to promote a prosocial classroom climate in primary schools, by enhancing students' intrinsic motivation through the fulfillment of the three basic psychological needs (autonomy, competence, and relatedness). Our findings showed that for the total sample, there were no significant effects for either primary or secondary outcomes at mid‐ and end‐of‐school year (T2 and T3). Only for a small subgroup of students who indicated the program had been optimally implemented (approximately a quarter of the entire sample), positive effects were found compared to students in the control schools for basic psychological needs and a prosocial classroom climate, as well as school well‐being, safety at school, and antisocial behavior—primarily at mid‐year (T2). Teachers who implemented the program reported a more positive attitude toward stimulating prosocial behavior and autonomy at the end of the school year (T3). When teachers indicated that they had implemented the program as intended, no significant effects were found.

No positive effects of the Dutch Meaningful Roles program were found in the entire sample, which is contrary to findings from similar intervention programs (e.g., Manzano‐Sánchez and Valero‐Valenzuela 2019; Mesurado et al. 2018; Sklad et al. 2012). It is possible that one or more components of the program were not effective, or that the fulfillment of basic psychological needs is not the working mechanism of the program. Another explanation for not finding positive effects could be that more time is needed for implementation. One school year may not be enough to find effects for an approach that is intended to become part of the class/school culture (compared to interventions with a fixed number of sessions or components). Previous research showed that program effects are larger the longer a program runs (Huitsing et al. 2020; Limber et al. 2018). A recommendation is therefore to extend evaluations beyond a school year in future research. The null results could also be due to the low variability of the scores of the variables (e.g., prosocial behavior), which may have limited the power to detect effects (e.g., Klocek et al. 2024). Furthermore, the moderate to high average stability coefficients over time suggest that outcomes were relatively stable, potentially accounting for limited program effects. Finally, another possible reason is that the program was not implemented as intended, which can be an important prerequisite for the effectiveness of an intervention (e.g., Durlak and DuPre 2008), which we also observed in our research. Several factors may influence this, related to, for example, providers (e.g., teacher competence, ability, or motivation), aspects of the support system (e.g., organizational functioning of the school, colleague support, high workload), or the responsiveness of the program (e.g., students' needs or motivation; Combs et al. 2022; Rojas‐Andrade and Bahamondes 2019; Waller et al. 2017). Further research is needed to determine which factors are important and what is needed for optimal implementation.

Our findings underscore the vital role of program fidelity in shaping the effectiveness of school‐based programs aimed at fostering prosocial classroom climates. This aligned with previous research on programs aimed at promoting SEL and reducing antisocial behavior (Durlak et al. 2011; Durlak and DuPre 2008; Haataja et al. 2014). Our findings indicate that this also holds for programs targeting basic psychological needs; to promote prosocial classrooms, all components must be carried out frequently and consistently. In the case of the Dutch Meaningful Roles, this includes giving students responsibility and autonomy through meaningful assignments (i.e., classroom roles), facilitating class meetings to discuss group dynamics and atmosphere, and encouraging students to recognize and appreciate each other's strengths and positive behaviors through structured compliments.

Interestingly, most effects of program fidelity were found in the short term, halfway through the program. This could be due to motivational problems during the course of the implementation, difficulty in keep providing attention to the components, or pressure from other tasks that had to be performed in the second half of the school year, such as exams and holidays—even in the subsample with ideal program fidelity. In the first half of the year, it was likely that teachers and students were most motivated and had the most attention to implementation, because they had recently completed the training. This is in line with the reported ideal program fidelity, where fewer students reported having roles and working with the roles (T2 68%, T3 61%), and fewer students and teachers reported having weekly class meetings (students: T2 51%, T3 43%; teachers: T2 44%, T3 36%) at T3 compared to T2.

Alternatively, most effects at T3 were on bullying (victimization and teacher attitudes), which was not the primary focus of the program. Effects on negative, persistent behaviors such as bullying may take longer to develop, since these processes develop mainly through complex group processes (Thornberg 2011). It could be that longer exposure to the program is mainly associated with effects on secondary outcomes. Finally, it is also possible that fewer effects at T3 were found because of lower power (*N* = 202 at T3 compared to *N* = 253 at T2).

### 4.1 Strengths and Limitations

There are several limitations of the study that need to be taken into account. Although we had a higher response rate than previous research (Tigges 2003; Wolfenden et al. 2009), approximately one‐third of the eligible sample did not participate in the study. Previous studies showed biased samples when using active compared to passive consent, such as more female and younger participants, fewer ethnically diverse students, and less problem behaviors (Courser et al. 2009; Liu et al. 2017; Pokorny et al. 2001). It is important that future research takes this into account to ensure that as many eligible participants as possible actually participate, and determines whether active consent is required, or whether passive consent is also an option. Another potential sample bias is that the study was conducted only in KiVa schools: schools that use KiVa as a social safety program. Although the program was designed for this population, it is unclear whether our results generalize to non‐KiVa schools (those that use social safety programs other than KiVa). Because all schools in the Netherlands are required to develop a safety plan to combat bullying (Safety at School Act), and several anti‐bullying programs have been found to be effective in the Netherlands (e.g., Kanjertraining, PRIMA; Netherlands Youth Institute 2025; Orobio De Castro and Mulder 2018; Vermande 2015), it is expected that most schools are comparable to KiVa schools. Future research should determine whether the Dutch Meaningful Roles also has positive effects on schools that implement other social safety programs. Despite these potential biases, the participating intervention and control schools were spread throughout the Netherlands, in both smaller villages and cities, suggesting that the sample is representative of a large part of the Dutch population.

Additionally, there were differences at baseline between the experimental and control condition, as well as between participants and dropouts, both among students and teachers. Even though we controlled for baseline differences between the experimental and control condition in the analyses, these differences suggest that the randomization was not entirely successful and that the internal validity of the study may have been weakened. For the analyses with program fidelity, students with high program fidelity generally reported higher and more positive scores than students with low program fidelity or students in the control group. It is possible that the positive effects can be explained by factors other than the program itself, for example, specific student characteristics such as higher intrinsic (prosocial) motivation or behavior. However, the baseline differences were small, aligning with previous research showing that higher baseline levels are associated with stronger effects (e.g., Stjerneklar et al. 2019).

Methodological limitations included our self‐constructed questionnaires and the relatively low power for both student and teacher analyses for program fidelity. While most of the instruments in this study had been validated and/or used in previous studies, this was not the case for every instrument (e.g., injunctive norm, teacher questionnaires). It is possible that the use of self‐constructed questionnaires resulted in earlier and/or larger positive effects. However, we also found positive effects on validated questionnaires, not just on self‐constructed instruments. In the analyses involving program fidelity, we had relatively low power, so the results must be interpreted with caution.

### 4.2 Practical Implications and Suggestions for Future Research

Our findings show that no positive effects were found for the Dutch Meaningful Roles in the entire sample compared to students in control schools. However, program fidelity does appear to be important for program effectiveness, as positive effects were observed for a subgroup of students with high program fidelity. Program fidelity is reported by few studies (Bruhn et al. 2015; Harn et al. 2013; van Loon et al. 2020), while it is essential for drawing correct conclusions about the effectiveness of a program. Developers of intervention programs and researchers investigating the effectiveness of such programs must recognize this, and are advised to focus and pay attention to correct implementation of the protocol. To enhance implementation of school‐based programs, previous research recommended that positive relationships between external facilitators and school staff should be created and maintained, the program should be appealing to key consumers of the program, the program should be visible, and adequate training opportunities should be provided for school staff (Gee et al. 2021; Kaufman and Huitsing 2019; van Loon et al. 2024; Wagner et al. 2004). Furthermore, most program effects were observed on the short term, halfway through the program. This indicates that it is important for program providers to be involved in the implementation throughout the whole implementation process (or longer), not just at the beginning.

A striking observation was that only a small percentage of students and teachers felt that the program was implemented as intended, approximately a quarter of the entire sample. It is necessary to better understand how the program can be optimally implemented and secured. The three main components were implemented reasonably well separately (reported ideal program integrity was 43%–68% and 36%–93% for students and teachers, respectively), but the combination of all three was not (18%–23% for students, 26%–34% for teachers). It appears that teachers implemented the program reasonably well, at least the individual components, but that combining these components and reaching all students in a class was difficult. It is therefore important to investigate how the program components are deployed (apart from frequency, as examined in the current study), what the working mechanisms are, and which sources of support schools need and which schools benefit most from this support (Gee et al. 2021; Kaufman and Huitsing 2019). It is also valuable to investigate whether components of Meaningful Roles – and the program as a whole—are also effective in schools that work with social safety programs other than KiVa.

Teachers in intervention schools reported positive effects on their attitudes toward stimulating prosocial behavior and autonomy compared to teachers in control schools. Although there may be some bias because teachers were not blinded to the fact that they participated in the intervention, it appears that teachers recognize the importance of the Dutch Meaningful Roles program, and thereby working in an autonomy‐enhancing way. Previous research demonstrated that if teachers apply a more autonomy‐supportive motivational style in their teaching, this has positive effects for both teachers and students, including greater job satisfaction and more student engagement in the classroom (Cheon et al. 2019). Because the teachers' role in this program—and most school‐based interventions—is crucial, it is essential that teachers support the program and are motivated to implement it. For example, previous research on a preventive intervention for young children showed that teachers who expressed more concerns about the intervention completed fewer program activities, while teachers who were more positive about their job, work environment, and principal and reported greater engagement completed more program activities (Baker et al. 2010).

## 5 Conclusion

Promoting a prosocial classroom climate is important because of its positive effects on children's academic and psychological well‐being and an optimal learning environment. The current study examined the effectiveness of a school‐based program, the Dutch Meaningful Roles program, aimed at promoting a prosocial classroom climate by enhancing basic psychological needs—autonomy, competence, and relatedness. Results across the entire sample showed no effects for students. Teachers reported a more positive attitude towards stimulating prosocial behavior and autonomy. When students perceived that the program was implemented as intended, these students fared better than students in control schools in terms of basic psychological need satisfaction, prosocial classroom climate, school well‐being and safety, and antisocial behavior. However, the effects were small and only found in a small group of students (approximately a quarter of the entire sample). It is important to understand the key conditions for optimal implementation, as this is a crucial condition for program effects to flourish.

## Author Contributions

**Amanda W. G. Loon:** conceptualization, formal analysis, project administration, visualization, writing – original draft, data curation, writing – review and editing, investigation, methodology. **Tessa M. L. Kaufman:** funding acquisition, conceptualization, supervision, writing – review and editing, methodology, project administration.

## Ethics Statement

The Ethical Committee of Utrecht University approved the design of this study (number 23‐0082). Written informed consent was obtained from children's legal guardian(s) and children older than 12 years. Additionally, teachers and school leaders gave written active informed consent for their own participation. All experiments were performed in accordance with relevant guidelines and regulations (such as the Declaration of Helsinki).

## Conflicts of Interest

The two authors (Amanda W. G. Loon and Tessa M. L. Kaufman) coordinated the implementation and evaluation of the Meaningful Roles (SterkWerk) program and are members of the steering committee. Program dissemination was done by a separate company (KiVa; www.sterkwerkschool.nl). The authors declare that they have no competing or conflicts of interest regarding this study.

## Supporting information

Supporting File

## Data Availability

The data that support the findings of this study are openly available in DANS at https://doi.org/10.17026/SS/1GJLI8. The data obtained in the current study are publicly available and are archived on the DANS Easy environment from the KNAW as agreed upon with NWO (open access policy). The corresponding author can be contacted for more information.
