# Supplementary material for: What Is Needed to Create a Prosocial Classroom? The Effectiveness of the Dutch Meaningful Roles Program in Children: A Cluster Randomized Controlled Study
Source: J Adolesc. 2026 Jun 5;98(6):1906–27. doi: 10.1002/jad.70188 (PMC13440710; doi:10.1002/jad.70188)
Supplement: Supplementary file 1 [file JAD-98-1906-s001.docx]

**Supplementary Material**

**Instruments**

***Primary outcomes***

*Prosocial behavior* was measured by several instruments measuring different domains. Individual prosocial behavior was measured with the subscale prosocial behavior of the Strengths and Difficulties Questionnaire (SDQ), Dutch version (Van Widenfelt et al., 2003), adapted for the current study. Four items were used (e.g., “I try to be kind to my classmates”), rated on a 4-point scale from 1 (*never*) to 4 (*always*). The SDQ-Dutch had sufficient psychometric properties in samples with children and young adolescents, with acceptable internal consistency, test-retest reliability, and good concurrent validity, with a Cronbach’s alpha of > 0.60 for the prosocial subscale (Muris et al., 2003; Van Widenfelt et al., 2003). Mean scores were computed, with higher scores reflecting more prosocial behavior (α = 0.63-0.69 for T1-T3).

Prosocial behavior as an injunctive norm (i.e., children’s attitudes towards prosocial behavior that are on average held in the classroom; Sentse et al., 2015) was assessed with three items specifically developed for this study (i.e., What can make you popular in your class?: “Helping each other”, “Being nice to each other” or “Working together”), rated on a 3-point scale from 1 (*not true*) to 3 (*definitely true*). Mean scores were computed, with higher scores reflecting a higher injunctive norm of the group towards prosocial behavior (α = 0.71-0.83 for T1-T3).

Cooperation and conflict in the classroom were measured with the Classroom Peer Context Questionnaire (CPCQ), subscales cooperation and conflict, respectively (Boor-Klip et al., 2014, 2016, 2017), rated on a 5-point scale ranging from 1 (*not true*) to 5 (*completely true*). The cooperation subscale consists of four items assessing the extent to which children experience positive behavior (e.g., “In this classroom, children help each other”). The conflict subscale consists of four items assessing the extent to which children experience negative behavior. For the current study, two items were used (e.g., “In this classroom, children are unkind to each other”). The CPCQ had adequate psychometric properties in a children sample – such as reliability, construct and concurrent validity, and stability – with a Cronbach’s alpha of > 0.79 for the cooperation and conflict subscales (Boor-Klip et al., 2014, 2016, 2017). Mean scores were computed, with higher scores reflecting more classroom cooperation (α = 0.74-0.81 for T1-T3) and more classroom conflict (α = 0.71-0.76 for T1-T3), respectively.

*Intrinsic (prosocial) motivation* was measured with four items developed for this study, based on a measure of social achievement goals (Rudolph et al., 2011; e.g., “I think it is important to help someone”), rated on a 3-point scale ranging from 1 (*not true*) to 3 (*definitely true*). Mean scores were computed, with higher scores reflecting more intrinsic (prosocial) motivation (α = 0.65-0.71 for T1-T3).

*Autonomy* was measured with items based on research on de Vreedzame School (Battistich et al., 1997; Lickona & Davidson, 2003; Pauw, 2013) and the Perceived Choice and Awareness Scale (Sheldon et al., 1996), which aimed to measure social responsibility and autonomy, respectively. Five items were used (e.g., “In this classroom, children help with making group appointments” or “In this classroom, you can choose how you want to do something”), rated on a 4-point scale ranging from 1 (*never*) to 4 (*always*). Mean scores were computed, with higher scores reflecting more autonomy (α = 0.67-0.74 for T1-T3).

*Competence* was measured with five items (e.g., “I think positively about myself”) based on the Rosenberg Self-Esteem Scale (RSES), Dutch version (Franck et al., 2008; Rosenberg, 1965), rated on a 3-point scale ranging from 1 (*not true*) to 3 (*definitely true*). The Dutch RSES showed high internal consistency in adults (Franck et al., 2008) and sufficient psychometric properties in adolescent samples, such as construct validity (Bagley, 1997; Cong & Cheong, 2022). Mean scores were computed, with higher scores reflecting more competence (α = 0.77-0.81 for T1-T3).

*Relatedness* was measured with items of the Classroom Peer Context Questionnaire (CPCQ), subscales comfort and cohesion (Boor-Klip et al., 2014, 2017), rated on a 5-point scale ranging from 1 (*not true*) to 5 (*completely true*). The comfort subscale consists of four items assessing the extent to which children feel at ease in the classroom (e.g., “In this classroom, I can be myself”). The cohesion subscale consists of four items assessing the extent to which the children in the classroom spend time together. For the current study, two items of this subscale were used (e.g., “In this classroom, everyone belongs to the group”). The CPCQ had adequate psychometric properties – such as reliability, construct and concurrent validity, and stability – in a children sample, with a Cronbach’s alpha > 0.68 for the comfort and cohesion subscale (Boor-Klip et al., 2014). A total mean score was computed, with higher scores reflecting more relatedness (α = 0.87-0.89 for T1-T3).

***Secondary outcomes***

*School wellbeing* was measured with seven items specifically developed for this study, based on research on KiVa (Kärnä et al., 2011; Kaufman et al., 2021), assessing feelings of enjoyment and connectedness to the classroom and school (7 items; e.g., “I like going to school”), rated on a 4-point scale ranging from 1 (*never*) to 4 (*always*). These items have been used in previous research examining the effectiveness of KiVa in the Netherlands, where high reliability was reported (Kaufman et al., 2021). A total mean score was computed, with higher scores reflecting more positive school wellbeing (α = 0.90-0.91 for T1-T3).

*Safety at school* was measured with one item developed for this study, assessing the sense of school safety (i.e., “I feel safe in and around school”), rated on a 5-point scale ranging from 1 (*very unsafe*) to 5 (*very safe*). A higher score reflects a higher sense of safety at school.

*Antisocial behavior* was measured by assessing bullying and bullying victimization with a selection of items of the Olweus Bully-Victim Questionnaire, Dutch version (OBVQ; (Olweus, 1996). Two questions were examined related to bullying (“How often have you bullied other children in the past few months?”) and bullying victimization (“How often have you been bullied in the past few months?”). Responses were rated on a 5-point Likert scale: *I did not bully/I was not bullied* (1), *once or twice* (2), *two or three times a month* (3), *about once a week* (4), and *several times a week* (5). Seven additional questions related to bullying victimization, and asked about behaviors that are associating with bullying (e.g., “I was threatened or forced to do things I did not want to do”), rated on 5-point Likert scale ranging from 1 (*not at all*) to 5 (*several times a week*). The OBVQ showed adequate psychometric properties in children samples – such as reliability, construct, and concurrent validity – with a Cronbach’s alpha above 0.80 (Gonçalves et al., 2016; Khawar, 2015; Kyriakides et al., 2006). Because we aimed to investigate general reductions in bullying victimization, we used the maximum score given on one of the specific items (Huitsing et al., 2020). Higher scores reflect more bullying (individual item) and bullying victimization (individual item and maximum score of 7 items: α = 0.89-0.88 for T1-T3).

*Teacher attitudes about bullying* (student perception) were measured with items based on research on KiVa (Veenstra et al., 2014). Three items concern discussing bullying (“Has your teacher ever mentioned bullying during class?”, with answer options: *no*, *once*, *2-4 times*, *5-8 times*, *more than 8 times*), attitude towards bullying (“What does your teacher think about bullying?”, with answer options: *it is a good thing*, *it does not matter*, *I do not know,* *it is a bad thing*, *it is a very bad thing*), and effectiveness to reduce bullying (“What can your teacher do to stop bullying?”, with answer options: *nothing*, *very little*, *a little*, *a lot*, *very much*). A total mean score was calculated, with higher scores reflecting more positive teacher attitudes towards bullying (α = 0.35-0.42 for T1-T3). Because the overall scale had very low reliability, we performed the analyses per item.

*Teacher attitudes towards stimulating basic psychological need fulfilment* were assessed by teachers, with specific questions developed for this study, related to the main components of Meaningful Roles. Teachers were asked what they consider important in their job as a teacher to stimulate, whether they actually stimulate this, whether they are good at it, and whether they have knowledge about it, rated on a scale of 1-10. Four subscales were created, for prosocial behavior (7 items, e.g., “I think it is important as a teacher to stimulate that children help each other”; α = .80-.85 for T1-T3), autonomy (5 items, e.g., “I am good at giving children responsibility”; α = .77-.78 for T1-T3), competence (4 items, e.g., “I make sure that children feel that they are good at something”; α = .82-.83 for T1-T3), and relatedness (7 items, e.g., “I know how to provide a safe atmosphere in my classroom, so that everyone is included”; α = .84-.86 for T1-T3). We used a factor analysis (PAF, principal axis factoring) to examine the extent to which the questionnaire indeed reflected four separate subscales. The KMO (Kaiser-Meyer-Olkin) was >.80. The Bartlett’s test of sphericity was significant (*p* < .001), and factor 1 explained between 50% and 67% of the variance for all subscales. PCA (principal component analysis) showed the same pattern. Because the individual subscales explained > 50% of the variance, the subscales had high internal consistency, and al factor loadings were sufficient (> .3), there was good support for the separate subscales for this study. Higher scores reflect a positive attitude towards stimulating prosocial behavior and basic psychological needs (autonomy, competence, and relatedness).

*Civic skills of students* were assessed by teachers, with specific questions developed for this study, based on the citizenship questionnaire (Daas et al., 2019)Four domains were assessed by asking teachers how the children in their class: 1) act democratically (3 items, e.g. “accept decisions made by the whole class (majority decisions); α = .70-.74 for T1-T3)”, 2) act socially responsible (2 items, e.g., “stand up for each other”; α = .54-.63 for T1-T3), 3) deal with conflicts (2 items, e.g., “consider the other person in a conflict when looking for a solution”; α = .71-.73 for T1-T3), and 4) deal with differences (3 items, e.g., “are curious about what other children are good at (qualities)”; α = .38-.54 for T1-T3). Responses were rated on a scale ranging from 1 (*(almost) never*) to 4 (*(almost) always*). Due to low reliability for some subscales, a total scale was created where higher scores reflect more positive civic skills of students (10 items, α = 0.81-0.85 for T1-T3). Factor analysis (both PAF and PCA) showed that the questionnaire consisted of a single factor (with >30% explained variance).

**Table S1**.

*Demographics at Baseline (T1) and Differences at T1 Between the Experimental (N = 1149) and Control (N = 1084) Condition for Children.*

|  | Experimental condition | Control condition | Differences at T1 |
| --- | --- | --- | --- |
|  | *M* (*SD*) | *M* (*SD*) | *t* (*df*) |
| Age (years)^a^ | 9.59 (1.20) | 9.51 (1.22) | -1.643 (2227) |
|  | *N* (%) | *N* (%) | χ2 (*df*) |
| Sex  Boy  Girl  Other or don’t know | 547 (47.6)  553 (48.1)  49 (4.3) | 534 (49.3)  516 (47.6)  34 (3.1) | 2.258 (2) |
| Gender  Boy  Girl  Other^b^ | 524 (45.6)  500 (43.5)  125 (10.9) | 503 (46.4)  449 (41.5)  131 (12.1) | 1.360 (2) |
| Ethnic identity  Western  Non-Western | 995 (87.9)  137 (12.1) | 937 (88.1)  127 (11.9) | 0.014 (1) |
| Grade^c^  5  6  7  8 | 210 (18.3)  287 (25.0)  324 (28.3)  325 (28.4) | 237 (21.9)  276 (25.5)  286 (26.4)  284 (26.2) | 5.197 (3) |

*Note*: ^a^: *N* = 2229 students reported their age (*N* = 1147 experimental condition, *N* = 1082 control condition). ^b^: Categories are other, boy and girl, no boy/girl, don’t know or don’t want to say. ^c^: N = 3 students reported to be in grade 4, all from the experimental condition. These students were not included in the analyses.

*p < .05, **p < .01, ***p < .001.

**Table S2**.

*Demographics at Baseline (T1) and Differences at T1 Between the Experimental (N = 115) and Control (N = 110) Condition for Teachers.*

|  | Experimental condition | Control condition | Differences at T1 |
| --- | --- | --- | --- |
|  | *M* (*SD*) | *M* (*SD*) | *t* (*df*) |
| Age (years) | 39.38 (10.75) | 37.95 (11.45) | -0.905 (194) |
|  | *N* (%) | *N* (%) | χ2 (*df*) |
| Sex  Male  Female  Don’t want to say | 11 (12.5)  76 (86.4)  1 (1.1) | 33 (32.7)  68 (67.3)  0 (0.0) | 11.605 (2)** |
| Gender  Male  Female  Don’t want to say | 11 (12.5)  77 (87.5)  0 (0.0) | 32 (31.7)  68 (67.3)  1 (1.0) | 10.972 (2)** |
| Ethnic identity  Western  Mix Western-non Western | 87 (100.0)  0 (0.0) | 98 (97.0)  3 (3.0) | 2.626 (1) |
| Grade  5-6  7-8  Other (e.g., 5-8, 6-8, 6-7) | 51 (44.3)  58 (50.4)  6 (5.2) | 48 (43.6)  48 (43.6)  14 (12.7) | 4.125 (2) |

*Note*: *p < .05, **p < .01, ***p < .001.

**Table S3**.

*Descriptives of Treatment Fidelity Questions for Children (Experimental Condition Only).*

|  | **T2** |  | **T3** |  |
| --- | --- | --- | --- | --- |
|  | ***N* (%)** | ***M* (*SD*)** | ***N* (%)** | ***M* (*SD*)** |
| Did you have a role the past few months?  I had no role  I had one role  I had two roles  I had three roles  I had four or more roles | 271 (24.4)  393 (35.4)  288 (25.9)  92 (8.3)  66 (5.9) | 2.36 (1.12) | 250 (22.4)  335 (30.1)  290 (26.0)  134 (12.0)  105 (9.4) | 2.56 (1.23) |
| How often were you working with the role(s)?  Multiple times a day  Once a day  Once a week  Once a month  Less than once a month  Never | 180 (21.3)  264 (31.3)  241 (28.6)  70 (8.3)  53 (6.3)  36 (4.3) | 2.60 (1.31) | 162 (18.7)  297 (34.3)  255 (29.4)  68 (7.8)  40 (4.6)  45 (5.1) | 2.61 (1.29) |
| Have you had any class meetings in the past few months?  No meeting  One meeting  One meeting a month  Two meetings a month  One meeting per week | 179 (16.2)  135 (12.2)  92 (8.2)  142 (12.8)  560 (50.5) | 3.69 (1.56) | 178 (16.0)  161 (14.5)  133 (12.0)  166 (14.9)  473 (42.6) | 3.54 (1.53) |
| I have received compliments from my classmates  Multiple times a day  Once a day  Once a week  Once a month  Less than once a month  Never | 145 (13.1)  190 (17.2)  322 (29.1)  166 (15.0)  128 (11.6)  155 (14.0) | 3.37 (1.56) | 148 (13.3)  191 (17.2)  316 (28.4)  173 (15.6)  166 (14.9)  117 (10.5) | 3.33 (1.52) |

**Table S4.**

Descriptives of Treatment Fidelity Questions for Teachers (Experimental Condition Only).

|  | **T2** |  | **T3** |  |
| --- | --- | --- | --- | --- |
|  | ***N* (%)** | ***M* (*SD*)** | ***N* (%)** | ***M* (*SD*)** |
| To what extent were the children able to carry out the roles independently?  Not at all independent  Not independent  Somewhat independent  Independent  Very independent | 1 (1.3)  0 (0.0)  19 (25.0)  40 (52.6)  16 (21.1) | 3.92 (0.76) | 0 (0.0)  0 (0.0)  16 (23.2)  33 (47.8)  20 (29.0) | 4.06 (0.73) |
| How many times has there been a meeting?  Never  Sometimes  Once a month  Twice a month  Every week  Multiple times a week | 6 (8.0)  5 (6.7)  7 (9.3)  24 (32.0)  33 (44.)  0 (0.0) | 3.97 (1.24) | 2 (2.9)  5 (7.2)  12 (17.4)  25 (36.2)  24 (34.8)  1 (1.4) | 3.97 (1.07) |
| How many times a week were the compliments given attention?  Never  Once a week  Multiple times a week  Once a day  Multiple times a day | 5 (6.8)  31 (41.9)  26 (35.1)  4 (5.4)  8 (10.8) | 2.72 (1.00) | 3 (4.3)  36 (52.2)  21 (30.4)  6 (8.7)  3 (4.3) | 2.57 (0.88) |
| How many compliments were given in class (approximately)?  Never  Once a week  Multiple times a week  Once a day  Multiple times a day | 3 (4.2)  24 (33.3)  29 (40.3)  4 (5.6)  12 (16.7) | 2.97 (1.11) | 3 (4.5)  21 (31.3)  30 (44.8)  1 (1.5)  12 (17.9) | 2.97 (1.11) |

**Table S5.**

*Differences at T1 for Demographics Between Participants and Dropouts at T2 and T3 for Students.*

|  | Participants | Drop-outs T2 and T3 | Differences at T1 |
| --- | --- | --- | --- |
|  | *M* (*SD*) | *M* (*SD*) | *t* (*df*) |
| Age (years) | 9.56 (1.20) | 9.28 (1.49) | 1.567 (73.01) |
|  | *N* (%) | *N* (%) | χ2 (*p*) |
| Sex  Boy  Girl  Other or don’t know | 1051 (48.6)  1032 (47.7)  79 (3.7) | 30 (42.3)  37 (52.1)  4 (5.6) | 1.574 (2) |
| Gender  Boy  Girl  Other | 995 (46.0)  916 (42.4)  250 (11.6) | 32 (45.1)  33 (46.5)  6 (8.5) | 0.867 (2) |
| Ethnic identity |  |  | 66.648 (1)*** |
| Western | 1893 (89.0) | 39 (56.5) |  |
| Non-Western | 234 (11.0) | 30 (43.5) |  |
| Grade  5  6  7  8 | 425 (19.7)  545 (24.5)  592 (27.4)  599 (27.7) | 22 (32.4)  18 (26.5)  18 (26.5)  10 (14.7) | 9.438 (3)* |
| *Note*. *p < .05, **p < .01, ***p < .001. | | | |

**Tabel S6.**

*Differences at T1 for Study Variables Between Participants (N = 2184) and Dropouts at T2 and T3 (N = 80) for Students.*

|  | Deelnemers | Dropouts op T2 en T3 | Verschillen op T1 |
| --- | --- | --- | --- |
|  | *M* (*SD*) | *M* (*SD*) | *t* (*df*) |
| Autonomy | 2.95 (0.53) | 2.85 (0.49) | 1.578 (2252) |
| Competence | 2.59 (0.42) | 2.51 (0.51) | 1.349 (79.72) |
| Relatedness | 4.13 (0.74) | 4.10 (0.73) | 0.390 (2252) |
| Intrinsic (prosocial) motivation | 2.66 (0.38) | 2.65 (0.41) | 0.263 (2256) |
| Prosocial behavior: individual | 2.84 (0.51) | 2.73 (0.58) | 1.910 (2258) |
| Prosocial behavior: injunctive norm | 2.49 (0.50) | 2.51 (0.43) | -0.291 (2262) |
| Prosocial behavior: cooperation | 3.96 (0.61) | 3.80 (0.59) | 2.189 (2257)* |
| Prosocial behavior: conflict | 2.14 (0.86) | 2.11 (0.84) | 0.274 (2258) |
| School wellbeing | 3.16 (0.63) | 3.30 (0.59) | -1.934 (2247) |
| Safety at school | 4.02 (0.93) | 3.99 (0.95) | 0.304 (2248) |
| Bullying | 1.21 (0.64) | 1.37 (0.83) | -1.613 (74.92) |
| Bullying victimization | 1.53 (1.04) | 1.61 (1.20) | -0.653 (2247) |
| Bullying victimization (max) | 1.86 (1.24) | 2.04 (1.40) | -1.209 (2244) |
| Teacher attitudes: discussing bullying | 2.53 (1.20) | 2.51 (1.38) | 0.152 (75.72) |
| Teacher attitudes: attitude towards bullying | 4.47 (0.95) | 4.40 (1.04) | 0.668 (2235) |
| Teacher attitudes: effectiveness to reduce bullying | 3.48 (1.07) | 3.74 (0.96) | -2.047 (2235)* |

*Noot.* *p < .05, **p < .01, ***p < .001.

**Table S7**.

*Differences at T1 Between Participants (N = 184) and Dropouts (N = 41) at T2 and T3 for Teachers.*

|  | Participants | Drop-outs T2 and T3 | Differences at T1 |
| --- | --- | --- | --- |
|  | *M* (*SD*) | *M* (*SD*) | *t* (*df*) |
| Age (years) | 38.87 (10.88) | 37.95 (12.02) | 0.461 (194) |
| Teacher attitudes: prosocial behavior | 7.90 (0.79) | 7.84 (0.73) | 0.391 (188) |
| Teacher attitudes: autonomy | 7.84 (0.92) | 7.74 (0.74) | 0.602 (188) |
| Teacher attitudes: competence | 8.06 (0.90) | 8.20 (0.71) | -0.837 (185) |
| Teacher attitudes: relatedness | 8.33 (0.79) | 7.38 (0.75) | -0.323 (185) |
| Civic skills of students | 3.03 (0.37) | 2.96 (0.35) | 0.788 (168) |
|  | *N* (%) | *N* (%) | χ2 (*df*) |
| Sex  Male  Female  Don’t want to say | 34 (20.6)  131 (79.4)  0 (0.0) | 10 (41.7)  13 (54.2)  1 (4.2) | 12.617 (2)** |
| Gender  Male  Female  Don’t want to say | 33 (20.0)  131 (79.4)  1 (0.6) | 10 (41.7)  14 (58.3)  0 (0.0) | 5.680 (2) |
| Ethnic identity  Western  Mix Western-non-Western | 163 (98.8)  2 (1.2) | 22 (95.7)  1 (33.3) | 1.264 (1) |
| Grade  5-6  7-8  Other (e.g., 5-8, 6-8, 6-7) | 86 (46.7)  83 (45.1)  15 (8.2) | 13 (31.7)  23 (56.1)  5 (12.2) | 3.198 (2) |
| Condition |  |  | 7.724 (1)** |
| Experimental condition  Control condition | 86 (46.7)  98 (53.3) | 29 (70.7)  12 (29.3) |  |

*Note*: *p < .05, **p < .01, ***p < .001.

**Correlation analyses**

Correlation analyses demonstrated that all primary outcomes (basic psychological needs and prosocial behavior) positively correlate to each other at T1-T3, except for conflict in the classroom, which is negatively related to all other outcomes (see Table S8 in the Supplementary Material). Regarding secondary outcomes, wellbeing and safety at school correlate positively with each other, and are negatively associated with bullying and bullying victimization at T1-T3, while bullying and bullying victimization are positively related to each other (see Table S9 in the Supplementary Material). In general for T1-T3, teacher attitudes about discussing bullying are not significantly associated with wellbeing and safety at school, and are positively associated with bullying and bullying victimization. Teacher attitudes towards bullying are positively associated with wellbeing, safety at school, and teacher attitudes about discussing bullying, but are negatively related to bullying and bullying victimization. Teacher attitudes regarding effectiveness in reducing bullying are positively related to wellbeing, safety at school, and teacher attitudes regarding discussing bullying and attitudes towards bullying, but are not significantly associated with bullying or bullying victimization (Table S9).

At T1, T2, and T3, basic psychological needs and prosocial classroom climate (i.e., prosocial behavior, wellbeing, safety, antisocial) positively correlate with each other, except for conflict, bullying, and bullying victimization, which are negatively related to these variables (see Tables S10-S12 in the Supplementary Material). Conflict, bullying, and bullying victimization are positively related to each other. Teacher attitudes regarding discussing bullying is positively related to autonomy (at T1, T3), intrinsic motivation (at T1, T2, T3), injunctive norm (at T1, T3), individual prosocial behavior (at T1, T2, T3), cooperation (at T3), conflict (at T1, T2), wellbeing (at T3), bullying (at T1, T2, T3) and bullying victimization (at T1, T2, T3), but negatively related to safety at school (at T2). Teacher attitudes toward bullying is positively associated with basic psychological needs, prosocial behavior, and wellbeing and safety at school, but negatively with conflict, bullying, and bullying victimization (at all three timepoints). Teacher attitudes regarding effectiveness in reducing bullying is positively related to basic psychological needs, prosocial behavior, and wellbeing and safety at school, but is not significantly associated with conflict (only at T2), bullying and bullying victimization (Tables S10-S12).

**Table S8.**

*Correlations Between Analyzed Variables (Primary Outcomes).*

|  | 1. | 2. | 3. | 4. | 5. | 6. | 7. | 8. | 9. | 10. | 11. | 12. | 13. | 14. | 15. | 16. | 17. | 18. | 19. | 20. | 21. | 22. | 23. |
| --- | --- | --- | --- | --- | --- | --- | --- | --- | --- | --- | --- | --- | --- | --- | --- | --- | --- | --- | --- | --- | --- | --- | --- |
| 1. Aut T1 | - |  |  |  |  |  |  |  |  |  |  |  |  |  |  |  |  |  |  |  |  |  |  |
| 2. Aut T2 | .466* | - |  |  |  |  |  |  |  |  |  |  |  |  |  |  |  |  |  |  |  |  |  |
| 3. Aut T3 | .416* | .534* | - |  |  |  |  |  |  |  |  |  |  |  |  |  |  |  |  |  |  |  |  |
| 4. Comp T1 | .319* | .243* | .220* | - |  |  |  |  |  |  |  |  |  |  |  |  |  |  |  |  |  |  |  |
| 5. Comp T2 | .236* | .359* | .262* | .579* | - |  |  |  |  |  |  |  |  |  |  |  |  |  |  |  |  |  |  |
| 6. Comp T3 | .242* | .276* | .356* | .527* | .632* | - |  |  |  |  |  |  |  |  |  |  |  |  |  |  |  |  |  |
| 7. Rel T1 | .535* | .429* | .401* | .494* | .401* | .395* | - |  |  |  |  |  |  |  |  |  |  |  |  |  |  |  |  |
| 8. Rel T2 | .378* | .572* | .454* | .370* | .535* | .415* | .632* | - |  |  |  |  |  |  |  |  |  |  |  |  |  |  |  |
| 9. Rel T3 | .335* | .458* | .596* | .334* | .409* | .527* | .580* | .707* | - |  |  |  |  |  |  |  |  |  |  |  |  |  |  |
| 10. Coop T1 | .543* | .414* | .386* | .292* | .229* | .235* | .602* | .443* | .411* | - |  |  |  |  |  |  |  |  |  |  |  |  |  |
| 11. Coop T2 | .386* | .551* | .453* | .256* | .329* | .270* | .503* | .635* | .519* | .551* | - |  |  |  |  |  |  |  |  |  |  |  |  |
| 12. Coop T3 | .353* | .449* | .575* | .201* | .262* | .337* | .458* | .555* | .655* | .493* | .638* | - |  |  |  |  |  |  |  |  |  |  |  |
| 13. Con T1 | -.327* | -.261* | -.260* | -.181* | -.150* | -.162* | -.438* | -.346* | -.315* | -.437* | -.345* | -.326* | - |  |  |  |  |  |  |  |  |  |  |
| 14. Con T2 | -.241* | -.308* | -.270* | -.173* | -.212* | -.195* | -.375* | -.499* | -.412* | -.329* | -.460* | -.406* | .506* | - |  |  |  |  |  |  |  |  |  |
| 15. Con T3 | -.246* | -.301* | -.381* | -.165* | -.163* | -.219* | -.376* | -.419* | -.495* | -.332* | -.429* | -.483* | .475* | .577* | - |  |  |  |  |  |  |  |  |
| 16. Inj T1 | .270* | .214* | .199* | .181* | .163* | .139* | .269* | .232* | .199* | .283* | .222* | .189* | -.139* | -.133* | -.129* | - |  |  |  |  |  |  |  |
| 17. Inj T2 | .260* | .332* | .303* | .162* | .217* | .194* | .298* | .371* | .325* | .260* | .362* | .318* | -.125* | -.189* | -.200* | .387* | - |  |  |  |  |  |  |
| 18. Inj T3 | .211* | .290* | .365* | .162* | .169* | .214* | .296* | .323* | .377* | .261* | .305* | .376* | -.160* | -.191* | -.231* | .356* | .526* | - |  |  |  |  |  |
| 19. Mot T1 | .338* | .261* | .235* | .235* | .137* | .131* | .339* | .271* | .230* | .345* | .288* | .236* | -.164* | -.148* | -.145* | .299* | .215* | .238* | - |  |  |  |  |
| 20 Mot T2 | .255* | .360* | .287* | .172* | .235* | .143* | .267* | .369* | .286* | .280* | .376* | .313* | -.131* | -.175* | -.173* | .224* | .315* | .280* | .495* | - |  |  |  |
| 21. Mot T3 | .227* | .303* | .408* | .136* | .179* | .241* | .265* | .308* | .396* | .264* | .322* | .410* | -.141* | -.160* | -.203* | .196* | .255* | .338* | .455* | .574* | - |  |  |
| 22. Pro T1 | .380* | .307* | .285* | .208* | .146* | .130* | .328* | .240* | .207* | .417* | .309* | .269* | -.178* | -.156* | -.177* | .287* | .214* | .204* | .452* | .377* | .331* | - |  |
| 23. Pro T2 | .296* | .416* | .352* | .165* | .234* | .170* | .299* | .364* | .300* | .342* | .474* | .365* | -.152* | -.217* | -.214* | .221* | .300* | .256* | .369* | .485* | .423* | .541* | - |
| 24. Pro T3 | .294* | .334* | .437* | .171* | .221* | .247* | .289* | .331* | .376* | .325* | .406* | .458* | -.157* | -.193* | -.222* | .185* | .259* | .319* | .334* | .403* | .503* | .488* | .624* |

Note. *p < .001. Aut = autonomy, Comp = competence, Rel = relatedness, Coop = cooperation in the classroom, Con = conflict in the classroom, Inj = injunctive norm, Mot = intrinsic (prosocial) motivation, Pro = individual prosocial behavior. Number of participants varies per variable and assessment (between *N* = 2140-2254).

**Table S9.**

*Correlations Between Analyzed Variables (Secondary Outcomes).*

|  | 1. | 2. | 3. | 4. | 5. | 6. | 7. | 8. | 9. | 10. | 11. | 12. | 13. | 14. | 15. | 16. | 17. | 18. | | 19. | | 20. | |
| --- | --- | --- | --- | --- | --- | --- | --- | --- | --- | --- | --- | --- | --- | --- | --- | --- | --- | --- | --- | --- | --- | --- | --- |
| 1. Well T1 | - |  |  |  |  |  |  |  |  |  |  |  |  |  |  |  |  |  | |  | |  | |
| 2. Well T2 | .649*** | - |  |  |  |  |  |  |  |  |  |  |  |  |  |  |  |  | |  | |  | |
| 3. Well T3 | .588*** | .695*** | - |  |  |  |  |  |  |  |  |  |  |  |  |  |  |  | |  | |  | |
| 4. Safe T1 | .405*** | .327*** | .314*** | - |  |  |  |  |  |  |  |  |  |  |  |  |  |  | |  | |  | |
| 5. Safe T2 | .339*** | .459*** | .377*** | .431*** | - |  |  |  |  |  |  |  |  |  |  |  |  |  | |  | |  | |
| 6. Safe T3 | .328*** | .382*** | .490*** | .371*** | .480*** | - |  |  |  |  |  |  |  |  |  |  |  |  | |  | |  | |
| 7. Bull T1 | -.183*** | -.183*** | -.145*** | -.171*** | -.173*** | -.175*** | - |  |  |  |  |  |  |  |  |  |  |  | |  | |  | |
| 8. Bull T2 | -.123*** | -.136*** | -.168*** | -.134*** | -.156*** | -.162*** | .314*** | - |  |  |  |  |  |  |  |  |  |  | |  | |  | |
| 9. Bull T3 | -.152*** | -.140*** | -.179*** | -.123*** | -.182*** | -.165*** | .291*** | .384*** | - |  |  |  |  |  |  |  |  |  | |  | |  | |
| 10. Vict T1 | -.332*** | -.272*** | -.261*** | -.266*** | -.246*** | -.264*** | .377*** | .230*** | .241*** | - |  |  |  |  |  |  |  |  | |  | |  | |
| 11. Vict T2 | -.265*** | -.336*** | -.291*** | -.238*** | -.317*** | -.272*** | .280*** | .375*** | .311*** | .522*** | - |  |  |  |  |  |  |  | |  | |  | |
| 12. Vict T3 | -.249*** | -.239*** | -.346*** | -.157*** | -.206*** | -.282*** | .248*** | .296*** | .449*** | .430*** | .556*** | - |  |  |  |  |  |  | |  | |  | |
| 13. TA1 T1 | .015 | .006 | -.008 | -.021 | -.038 | -.034 | .072*** | .047* | .080*** | .087*** | .042 | .066*** | - |  |  |  |  |  | |  | |  | |
| 14. TA1 T2 | .009 | .013 | -.008 | .004 | -.055** | -.017 | .048* | .084*** | .071*** | .022 | .048* | .063*** | .328*** | - |  |  |  |  | |  | |  | |
| 15. TA1 T3 | .010 | .024 | .042* | .005 | -.028 | .008 | .041 | .083*** | .082*** | .024 | .040 | .084*** | .309*** | .453*** | - |  |  |  | |  | |  | |
| 16. TA2 T1 | .115*** | .087*** | .084*** | .090*** | .084*** | .093*** | -.106*** | -.120*** | -.090*** | -.098*** | -.113*** | -.085*** | .164*** | .079*** | .023 | - |  |  | |  | |  | |
| 17 TA2 T2 | .155*** | .172*** | .173*** | .112*** | .106*** | .108*** | -.089*** | -.145*** | -.165*** | -.113*** | -.165*** | -.131*** | .086*** | .187*** | .093*** | .306*** | - |  | |  | |  | |
| 18. TA2 T3 | .127*** | .171*** | .191*** | .087*** | .126*** | .128*** | -.099*** | -.078*** | -.083*** | -.113*** | -.109*** | -.122*** | .098*** | .132*** | .201*** | .316*** | .407*** | - |  | |  | |  |
| 19. TA3 T1 | .111*** | .135*** | .090*** | .104*** | .067** | .024 | -.014 | -.015 | .029 | .029 | .008 | .042 | .160*** | .099*** | .094*** | .126*** | .091*** | .110*** | | - | |  | |
| 20. TA3 T2 | .111*** | .158*** | .150*** | .102*** | .078*** | .037 | .028 | .019 | .035 | -.008 | .006 | .010 | .079*** | .142*** | .121*** | .002 | .104*** | .080*** | | .465*** | | - | |
| 21. TA3 T3 | .100*** | .132*** | .144*** | .061** | .018 | .064** | .011 | .035 | .030 | -.003 | .046* | .012 | .089*** | .125*** | .231*** | .020 | .038 | .156*** | | .399*** | | .505*** | |

Note. **p* < .05, ***p* < .01, ****p* < .001. Well = school wellbeing, Safe = safety at school, Bull = bullying, Vict = bullying victimization, TA1 = teacher attitudes: discussing bullying, TA2 = teacher attitudes: attitudes toward bullying, TA3 = teacher attitudes: effectiveness in reducing bullying. Number of participants varies per variable and assessment (between *N* = 2120-2249).

**Table S10.**

*Correlations Between Analyzed Variables at T1.*

|  | 1. | 2. | 3. | 4. | 5. | 6. | 7. | 8. | 9. | 10. | 11. | 12. | 13. | 14. |
| --- | --- | --- | --- | --- | --- | --- | --- | --- | --- | --- | --- | --- | --- | --- |
| 1. Autonomy | - |  |  |  |  |  |  |  |  |  |  |  |  |  |
| 2. Competence | .319*** | - |  |  |  |  |  |  |  |  |  |  |  |  |
| 3. Relatedness | .535*** | .494*** | - |  |  |  |  |  |  |  |  |  |  |  |
| 4. Motivation | .338*** | .235*** | .339*** | - |  |  |  |  |  |  |  |  |  |  |
| 5. Inj. norm | .270*** | .181*** | .269*** | .299*** | - |  |  |  |  |  |  |  |  |  |
| 6. Prosocial beh. | .380*** | .208*** | .328*** | .452*** | .287*** | - |  |  |  |  |  |  |  |  |
| 7. Cooperation | .543*** | .292*** | .602*** | .345*** | .283*** | .417*** | - |  |  |  |  |  |  |  |
| 8. Conflict | -.327*** | -.181*** | -.438*** | -.164*** | -.139*** | -.178*** | -.437*** | - |  |  |  |  |  |  |
| 9. Wellbeing | .505*** | .475*** | .771*** | .360*** | .264*** | .356*** | .526*** | -.377*** | - |  |  |  |  |  |
| 10. Safety | .230*** | .244*** | .356*** | .133*** | .125*** | .138*** | .266*** | -.213*** | .405*** | - |  |  |  |  |
| 11. Bullying | -.141*** | -.169*** | -.126*** | -.119*** | -.074*** | -.144*** | -.128*** | .251*** | -.183*** | -.171*** | - |  |  |  |
| 12. Victimization | -.200*** | -.224*** | -.361*** | -.017 | -.068** | -.015 | -.191*** | .366*** | -.332*** | -.266*** | .377*** | - |  |  |
| 13. TA: discussing | .043* | -.027 | -.034 | .079*** | .042* | .056** | -.022 | .076*** | .015 | -.021 | .072*** | .087*** | - |  |
| 14. TA: atttitude | .159*** | .097*** | .120*** | .133*** | .083*** | .125*** | .153*** | -.130*** | .115** | .090*** | -.106*** | -.098*** | .164*** | - |
| 15. TA: effectiveness | .098*** | .058** | .113*** | .120*** | .070*** | .077*** | .101*** | -.045* | .111*** | .104*** | -.014 | .029 | .160*** | .126*** |

Note. **p* < .05, ***p* < .01, ****p* < .001. Inj. norm = injunctive norm, Prosocial beh. = prosocial behavior, Wellbeing = school wellbeing, Safety = safety at school, Victimization = bullying victimization, TA: discussing = teacher attitudes: discussing bullying, TA: attitude = teacher attitudes: attitudes toward bullying, TA: effectiveness = teacher attitudes: effectiveness in reducing bullying. Number of participants varies per variable and assessment (between *N* = 2234-2264).

**Table S11.**

*Correlations Between Analyzed Variables at T2.*

|  | 1. | 2. | 3. | 4. | 5. | 6. | 7. | 8. | 9. | 10. | 11. | 12. | 13. | 14. |
| --- | --- | --- | --- | --- | --- | --- | --- | --- | --- | --- | --- | --- | --- | --- |
| 1. Autonomy | - |  |  |  |  |  |  |  |  |  |  |  |  |  |
| 2. Competence | .359*** | - |  |  |  |  |  |  |  |  |  |  |  |  |
| 3. Relatedness | .572*** | .535*** | - |  |  |  |  |  |  |  |  |  |  |  |
| 4. Motivation | .360*** | .235*** | .369*** | - |  |  |  |  |  |  |  |  |  |  |
| 5. Inj. norm | .332*** | .217*** | .371*** | .315*** | - |  |  |  |  |  |  |  |  |  |
| 6. Prosocial beh. | .416*** | .234*** | .364*** | .485*** | .300*** | - |  |  |  |  |  |  |  |  |
| 7. Cooperation | .551*** | .329*** | .635*** | .376*** | .362*** | .474*** | - |  |  |  |  |  |  |  |
| 8. Conflict | -.308*** | -.212*** | -.499*** | -.175*** | -.189*** | -.217*** | -.460*** | - |  |  |  |  |  |  |
| 9. Wellbeing | .544*** | .501*** | .796*** | .399*** | .327*** | .417*** | .548*** | -.411*** | - |  |  |  |  |  |
| 10. Safety | .273*** | .284*** | .410*** | .173*** | .132*** | .195*** | .314*** | -.269*** | .459*** | - |  |  |  |  |
| 11. Bullying | -.085*** | -.109*** | -.153*** | -.063** | -.018 | -.127*** | -.130*** | .226*** | -.136*** | -.156*** | - |  |  |  |
| 12. Victimization | -.181*** | -.181*** | -.373*** | -.053* | -.070** | -.045* | -.234*** | .411*** | -.336*** | -.317*** | .375*** | - |  |  |
| 13. TA: discussing | .022 | -.006 | -.019 | .051* | .040 | .045* | -.011 | .104*** | .013 | -.055* | .084*** | .048* | - |  |
| 14. TA: atttitude | .182*** | .121*** | .182*** | .178*** | .079*** | .131*** | .149*** | -.127*** | .172** | .106*** | -.145*** | -.165*** | .187*** | - |
| 15. TA: effectiveness | .116*** | .109** | .164*** | .178*** | .160*** | .111*** | .131*** | -.038 | .158*** | .078*** | .019 | .006 | .142*** | .104*** |

Note. **p* < .05, ***p* < .01, ****p* < .001. Inj. norm = injunctive norm, Prosocial beh. = prosocial behavior, Wellbeing = school wellbeing, Safety = safety at school, Victimization = bullying victimization, TA: discussing = teacher attitudes: discussing bullying, TA: attitude = teacher attitudes: attitudes toward bullying, TA: effectiveness = teacher attitudes: effectiveness in reducing bullying. Number of participants varies per variable and assessment (between *N* = 2204-2219).

**Table S12.**

*Correlations Between Analyzed Variables at T3.*

|  | 1. | 2. | 3. | 4. | 5. | 6. | 7. | 8. | 9. | 10. | 11. | 12. | 13. | 14. |
| --- | --- | --- | --- | --- | --- | --- | --- | --- | --- | --- | --- | --- | --- | --- |
| 1. Autonomy | - |  |  |  |  |  |  |  |  |  |  |  |  |  |
| 2. Competence | .356*** | - |  |  |  |  |  |  |  |  |  |  |  |  |
| 3. Relatedness | .596*** | .527*** | - |  |  |  |  |  |  |  |  |  |  |  |
| 4. Motivation | .408*** | .241*** | .396*** | - |  |  |  |  |  |  |  |  |  |  |
| 5. Inj. norm | .365*** | .214*** | .377*** | .338*** | - |  |  |  |  |  |  |  |  |  |
| 6. Prosocial beh. | .437*** | .247*** | .376*** | .503*** | .319*** | - |  |  |  |  |  |  |  |  |
| 7. Cooperation | .575*** | .337*** | .655*** | .410*** | .376*** | .458*** | - |  |  |  |  |  |  |  |
| 8. Conflict | -.381*** | -.219*** | -.495*** | -.203*** | -.231*** | -.222*** | -.483*** | - |  |  |  |  |  |  |
| 9. Wellbeing | .583*** | .510*** | .802*** | .434*** | .347*** | .426*** | .571*** | -.414*** | - |  |  |  |  |  |
| 10. Safety | .276*** | .320*** | .436*** | .208*** | .170*** | .200*** | .333*** | -.277*** | .490*** | - |  |  |  |  |
| 11. Bullying | -.104*** | -.109*** | -.190*** | -.074*** | -.004 | -.082*** | -.137*** | .291*** | -.179*** | -.165*** | - |  |  |  |
| 12. Victimization | -.176*** | -.221*** | -.381*** | -.060** | -.079*** | -.037 | -.228*** | .385*** | -.346*** | -.282*** | .449*** | - |  |  |
| 13. TA: discussing | .089*** | -.021 | .038 | .117*** | .082*** | .099*** | .062** | .042 | .042* | .008 | .082*** | .084*** | - |  |
| 14. TA: atttitude | .179*** | .138*** | .184*** | .169*** | .099*** | .158*** | .195*** | -.131*** | .191*** | .128*** | -.083*** | -.112*** | .201*** | - |
| 15. TA: effectiveness | .178*** | .091*** | .154*** | .174*** | .157*** | .163*** | .137*** | -.066** | .144*** | .064** | .030 | .012 | .231*** | .156*** |

Note. **p* < .05, ***p* < .01, ****p* < .001. Inj. norm = injunctive norm, Prosocial beh. = prosocial behavior, Wellbeing = school wellbeing, Safety = safety at school, Victimization = bullying victimization, TA: discussing = teacher attitudes: discussing bullying, TA: attitude = teacher attitudes: attitudes toward bullying, TA: effectiveness = teacher attitudes: effectiveness in reducing bullying. Number of participants varies per variable and assessment (between *N* = 2206-2225).
